# Supramolecular assembly of novel achatin structurally similar peptide as potential G protein-coupled receptors anticancer ligands: focus on its pseudo-macrocycle nature

**DOI:** 10.1098/rsos.251026

**Published:** 2025-09-24

**Authors:** Joanna Bojarska, Martin Breza, Ingrid Jelemenska, Izabela Madura, Sepideh Jafari, Krzysztof Kaczmarek, Zyta Ziora, Wojciech Wolf

**Affiliations:** ^1^ Department of Chemistry, Institute of Inorganic and Ecological Chemistry, Technical University of Lodz, Lodz, Poland; ^2^ STU, Physical Chemistry, Bratislava, SK, Slovak Technical University, Bratislava, Slovakia; ^3^ Department of Chemistry, Technical University of Warsaw, Warsaw, Poland; ^4^ Institute of Organic Chemistry, Chemistry Department, Technical University of Lodz, Poland; ^5^ Department of Molecular Bioscience, University of Queensland, Brisbane, Queensland, Australia

**Keywords:** ultra-short peptides, G protein-coupled receptors, cancer, pseudo-macrocycle, density functional theory, supramolecular assembly

## Abstract

This study presents the synthesis and comprehensive characterization of a novel, modified ultra-short peptide, Ac-Phe-Aib-Deg-OH (*N*-acetyl-*L*-phenylalanyl-α-aminoisobutyryl*-*α,α-diethylglycine), (**1**), highlighting its potential as an anticancer ligand for G protein-coupled receptors (GPCRs). The structure of the compound was elucidated using single-crystal X-ray crystallography, revealing its pseudo-macrocyclic nature. Hydrogen bonds and dispersion forces drive the hierarchical supramolecular self-assembly of (**1**). A comparative analysis with structurally similar structures, that is, achatin derived from the Cambridge Structural Database, was conducted using diverse *in silico* methods. A detailed analysis of intra- and intermolecular interactions in the structures, especially using modern structural database-driven techniques, provides further insight into the preferential bonding mode of (**1**) as a potential drug candidate. Density functional theory calculations were performed to rationalize the interactions and reactivity of the molecules. To validate the predicted anticancer activity of the analysed molecules, we evaluated the binding affinities of these molecules to cancer-related GPCRs. We examined the interaction maps using molecular docking analysis with targets associated with various types of cancer. These findings suggest that (**1**) could be a promising candidate for future studies on designing innovative pseudo-macrocyclic ultra-short peptides containing unnatural amino acids for new targeted anticancer therapy.

## 1. Introduction

Combining the advantages of small traditional molecules and biologics, short peptides have unique features and better bio-pharmacy profiles than their longer analogues [1]. Structural modifications, such as cyclization or incorporation of unnatural amino acids (including amino acid analogues/mimetics, synthetic/*N*-methyl/*d*-amino acids, or *N*-substituted glycines), help overcome the limitations of peptides. Recently, macrocyclization has garnered increasing attention as an appealing strategy for enhancing the bio-pharmacological properties of peptides [2,3]. A macrocycle is a cyclic molecule with at least nine members (and three donors) [4]. It has a more rigid conformation that facilitates stronger binding to protein targets [5]. Macrocyclic peptide structures contain one or more rings and multiple amino acid residues [6]. Macrocyclo-peptides, neither too big nor too small, are called ‘a new Goldilocks drug class’ suitable for ‘undruggable’ targets. However, macrocyclic peptides, often containing 4–24 amino acids, fall between small-molecule drugs (‘beyond the rule of 5’). Thus, low oral bioavailability or cell permeability may be problematic. Pseudo-macrocycles that adopt a macrocyclic-like conformation via non-covalent intramolecular interactions [7] have improved lipophilicity and solubility. It offers advantages similar to true macrocycles, that is high stability, selectivity and binding affinity. Molecules with pseudorings may, among other things, change their conformation while passing through the cell. In other words, such chameleon-like molecules can switch between ‘open’ and ‘closed’ forms to achieve cell permeability.

Although ultra-short peptides, that is, tripeptides, are not typically classified as traditional macrocycles, they can exhibit certain macrocycle-like behaviours. It enables the formation of pseudo-macrocycles (when structural constraints and non-covalent interactions induce a cyclic-like conformation).

The pseudo-ring methodology, which monitors conformation via non-covalent (supramolecular) interactions, is relevant in drug design and the development of biomaterials with controllable properties [8]. Non-covalent interactions encompass intra- and intermolecular hydrogen bonding, π-based contacts, dispersion forces and hydrophobic interactions. Among these, hydrogen bonds are the most important in directing and regulating the forces of supramolecular assembly in bio-organic compounds [9,10].

It is worth noting that self-assembly is a common phenomenon in nature. Proteins and peptides associate to form diverse structures [11]. However, it should be emphasized that the association of even very large peptides is driven only by a few key interacting amino acid residues. Therefore, designing small self-assembled peptides is essential for understanding the factors that determine the self-(re)organization.

Ultra-short peptides provide tunable features by substituting for individual amino acid residues [12–14]. They may serve as templates for designing bioinspired materials or for studying self-assembly mechanisms.

Self-assembly leads to the formation of hierarchically organized supramolecular architectures that can be engineered at different levels of self-association. It induces new features through cooperative intermolecular interactions [15].

Developing short peptides that self-assemble into supramolecular helices or beta-sheets is a highly active area of research. The example of such a small peptide is achatin.

This is a tetrapeptide, H-Gly-Phe-Ala-Asp-OH, isolated from the ganglia of the African giant snail *Achatina fulica* [16]. Achatin-I (H-Gly-*D*-Phe-Ala-Asp-OH) has a turn conformation in both the solid and liquid states [17]. It has a neuroexcitatory effect [18] and cardiac activity [19] as well as antibacterial and analgesic properties [20]. It has also been tested for anti-inflammatory function [21]. More importantly, a noteworthy observation was that the pseudo-macrocycle nature of achatin and achatin-based peptides has potential as anticancer agents [22].

According to the World Health Organization, cancer is the leading cause of mortality worldwide. Despite the diverse treatment options, there is a pressing need to discover new drugs with reduced toxicity, enhanced effectiveness and growing demand for the development of new drug targets with high specificity.

G protein-coupled receptors (GPCRs) are among the most prominent families of membrane proteins. They are key regulators of various physiological processes, including cell signalling, metabolism, immune responses and homeostasis [23].

Among the GPCR superfamily, class A (rhodopsin-like) GPCRs are the most diverse and well characterized. Accumulating evidence suggests that class A GPCRs play a role in promoting cancer progression, metastasis, angiogenesis, immune evasion and drug resistance. Their accessibility as membrane proteins and compatibility with modulation by small molecules, peptides and biologics makes them appealing therapeutic targets for cancer treatment [24].

Several class A GPCRs have been implicated in tumour biology such as proliferation, survival and invasion [25]. The C-X-C motif chemokine receptor 4 (CXCR4) in class A is highly expressed in various cancers, including breast, lung and pancreatic cancer and facilitates metastasis through its ligand C-X-C motif chemokine (CXCL12) (Stromal cell-Derived Factor 1-alpha (SDF-1α)) [26–28]. C-C motif chemokine receptor 5 (CCR5) is involved in tumour invasion and immune evasion, especially in breast and colorectal cancers [29]. Likewise, formyl peptide receptor 1 (FPR1) facilitates inflammation-mediated angiogenesis and immune regulation in gastric and colorectal cancers [30]. Other notable receptors include protease-activated receptor 1 (PAR1), which induces tumour invasion and metastasis [31], and lysophosphatidic acid receptor 1 (LPA1), which plays a role in tumorigenesis and chemoresistance [32]. Moreover, sphingosine−1-phosphate receptor 1 (S1PR1), prostaglandin E2 receptor 4, thromboxane A2 receptor (TBXA2R), beta−2 adrenergic receptor (ADRB2) and opioid receptors (delta and mu opioid receptors) have been found to play significant roles in cancer progression. These receptors convey oncogenic signals through downstream signalling pathways, such as PI3K/AKT, MAPK/ERK, JAK/STAT and Rho GTPase pathways, thereby regulating tumour growth, immune evasion, metastasis and chemoresistance [33–37]. In recent decades, computational techniques have revolutionized the discovery of GPCR drugs.

Molecular *in silico* docking enables the prediction of interactions between receptors and ligands, as well as their binding affinities, thereby accelerating the screening of potential therapeutic leads [38].

In this article, within the context of the importance of investigation of small peptide (pseudo)macrocycles for innovative anticancer drugs, we report on the synthesis and complex supramolecular and docking analysis of modified ultra-short peptide, Ac-Phe-Aib-Deg-OH (International Union of Pure and Applied Chemistry (IUPAC) name: *N*-acetyl-*L*-phenylalanyl-α-aminoisobutyryl*-*α,α-diethylglycine) here called (**1**), highlighting its potential as an anticancer ligand of GPCRs.

**Figure 1.**
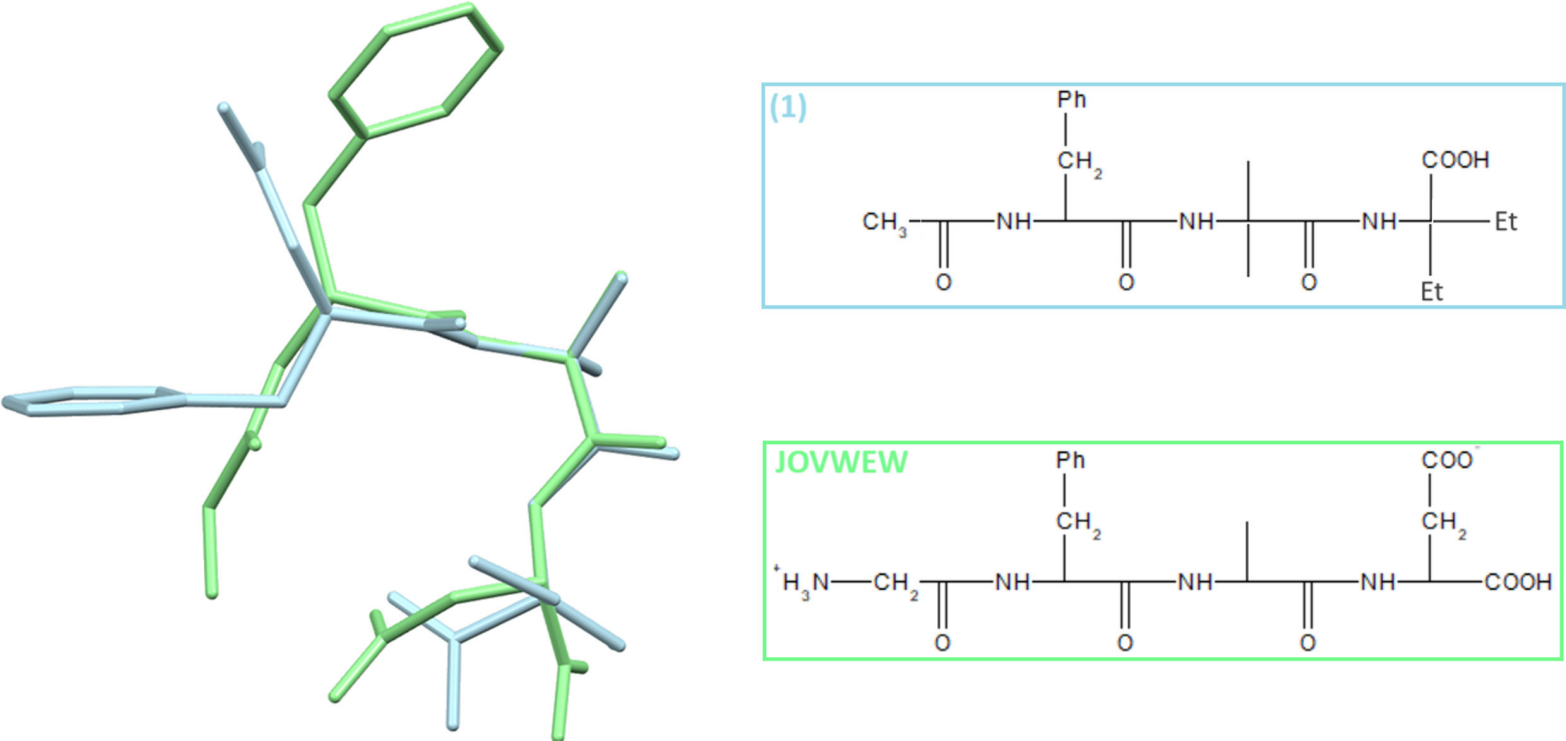
Superimposition of (**1**) and JOVWEW.

Structural modifications of (**1**) by the incorporation of unnatural amino acids (Aib—α-aminoisobutyric acid and Deg—α,α-diethylglycine) were hypothesized to increase the efficacy of peptide molecules and their interactions with protein targets. We also expected that Aib would provide a turn structure and subunit for H-bonding self-assembly. The crystal structure was solved using a single-crystal X-ray diffraction method, which, together with an extended Hirshfeld surface (HS) study, confirmed the relevance of H-bonding interactions in driving and stabilizing self-assembly. We report herein a comparative analysis of (**1**) with four related structures adopting a similar folded conformation (and pseudo-macrocycle nature) derived from the Cambridge Structural Database (CSD), i.e. such as achatin-I (Gly-*D*-Phe-Aib-Leu), CSD reference code JOVWEW [16], that is the most structurally similar to (**1**), see figure 1 and the electronic supplementary material, figure S1, *N*-acetyltyrosylleucylaspartic acid monohydrate (Tyr-Leu-Asp), BEFVEQ01 [39], *t*-butyloxycarbonyl-glycyl-phenylalanyl-a-isobutyryl-leucine methyl ester (Gly-Phe-Aib-Leu), HOTYAR [40] and methyl *N*-{2-[(benzene carbonyl)amino]-3-[4-(diphenylamine)phenyl]acryloyl}-3-(2,3-dihydro-1,4-benzodioxin-6-yl)alanylphenylalanylphenylalaninate dichloromethane solvate (Tyr-Tyr-Phe-Phe), VISYII [41], as a continuation of our ongoing studies of the exploration of supramolecular systems of peptide-based structures in terms of their potential bio-activity [42–49]. A detailed analysis of intra- and intermolecular interactions in the crystal structures of the peptide, especially using modern database-driven techniques, provides further insight into its preferential bonding mode as a potential drug candidate. Density functional theory (DFT) calculations were performed to rationalize the interactions and reactivity of the molecules. To validate the predicted anticancer activity of the analysed molecules, we evaluated the binding affinities of these molecules to cancer-related GPCRs. We examined the interaction maps via molecular docking analysis with the targets associated with diverse types of cancer.

This study explains the (supra)molecular and functional properties of a novel ultra-short peptide with a pseudo-macrocycle nature, providing more details on its potential drug ability and paving the way to design more optimized molecules for new smart targeted anticancer therapies.

## 2. Results and discussion

### 2.1. Single-crystal structure X-ray diffraction analysis

**Figure 2.**
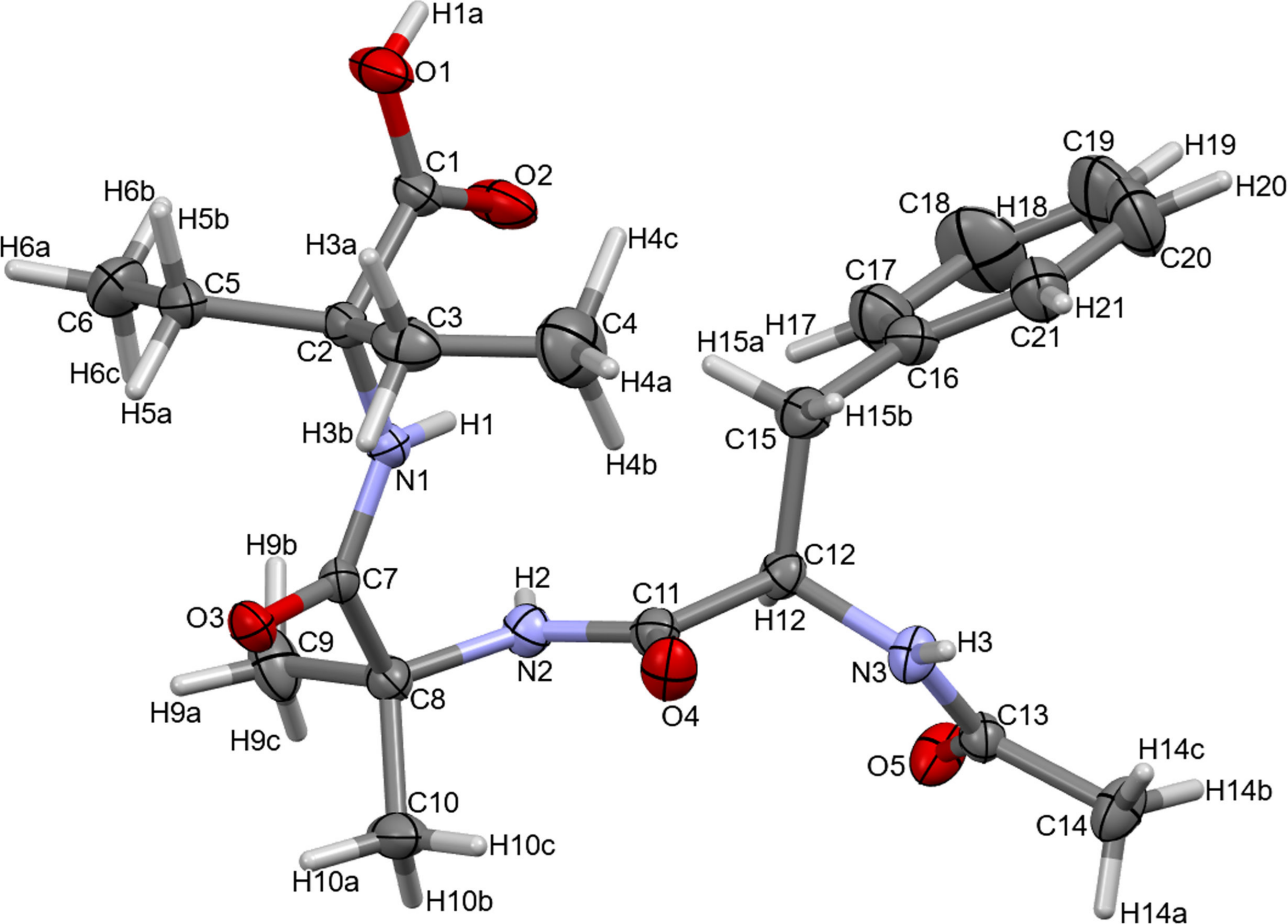
Molecular structure of (**1**), showing 50% probability thermal ellipsoids.

The synthesized compound (**1**) crystallizes in the orthorhombic crystal system with the space group *P*2_1_2_1_2_1_ with four molecules in the unit cell (Z = 4) and one in the asymmetric unit (*Z*′ = 1). The molecule of (**1**) exists in a neutral, non-ionized form. The three amide bonds are in a *trans* orientation. The phenyl ring is planar. The torsion angle between the phenyl and peptide plane (C16-C15-C12-N3) is 66.61^o^. The dihedral angles C7-C8-N2-C11 and C15-C12-C11-N2 are 66.15^o^ and 93.85^o^, respectively. Thus, the molecule of (**1**) adopts a folded conformation. Similar conformations were also observed in the four relevant structures retrieved from CSD. The molecular structure of (**1**) is depicted in figure 2, while the molecular structures of the other analysed compounds are presented in the electronic supplementary material, figure S2. More specifically, these structures adopt a beta-turn conformation. It can be noted that the turn conformation of JOVWEW reflects an essential feature in terms of the bioactivity of achatin-I [16]. It is worth noting that these structural insights also have relevance in understanding the dynamics of the binding of analysed molecules to target proteins (see section below).

**Figure 3.**
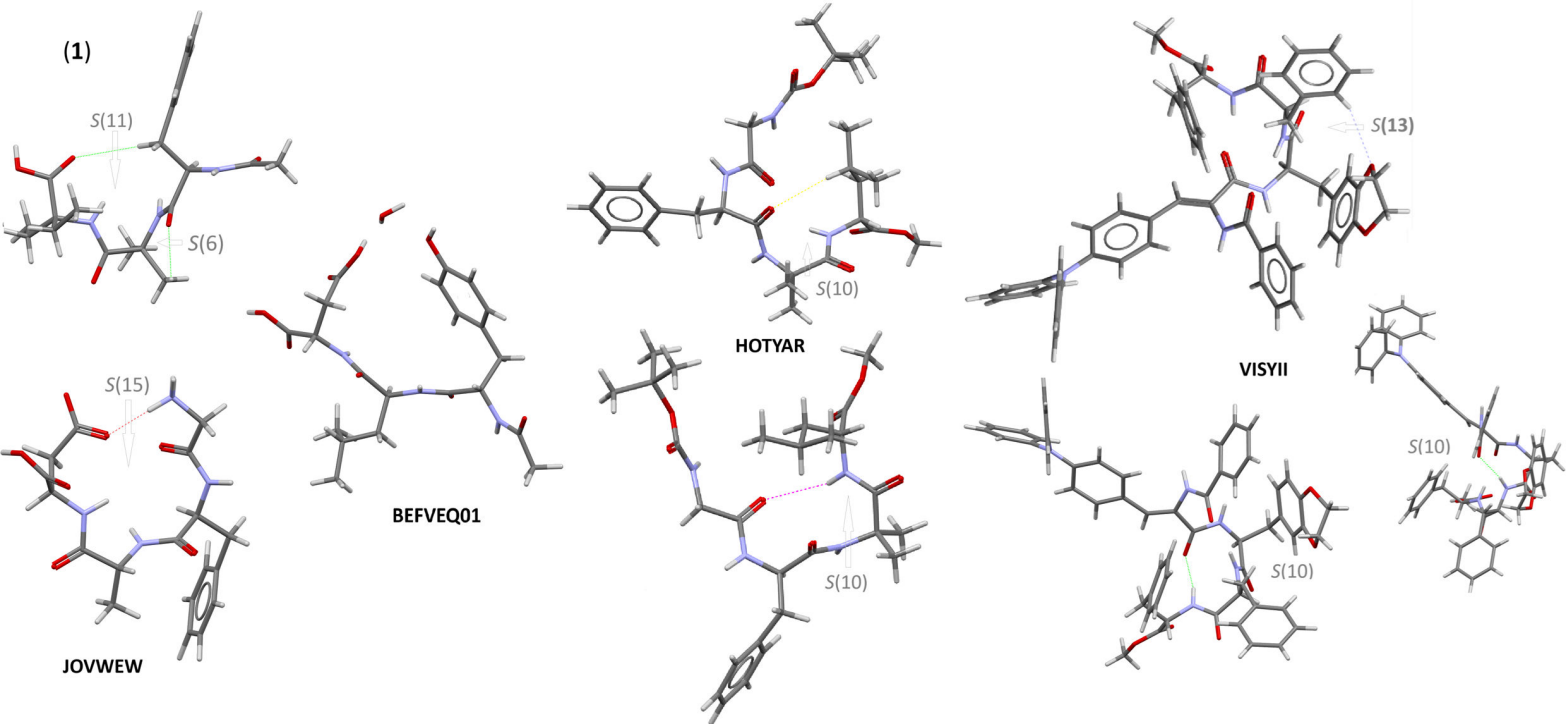
Molecular structures of analysed molecules with pseudo-macrocyclic nature, showing intramolecular H-bonding motifs.

A thorough comparative analysis of structurally similar structures derived from CSD revealed that intramolecular interactions stabilize the folded conformation, that is, N-H^…^O (in JOVWEW, HOTYAR and VISYII) and C-H^…^O (in (**1**), HOTYAR and VISYII), thus forming a pseudo-macrocyclic structure. From a supramolecular point of view, it can be described as ‘S’ (self) motifs according to the graph set notation [50] (figure 3). In JOVWEW, such motifs are formed by N-H^…^O, in (**1**) by C-H^…^O, while in HOTYAR and VISYII, either by N-H^…^O or C-H^…^O interactions. The exception is BEFVEQ01—despite its pseudo-macrocyclic nature—the intra-cyclic motif was not observed (the water molecule is a part of the crystal structure).

Electronic supplementary material, table S1 summarizes the respective lattice parameters and other crystallographic information, including refinement details, for either (**1**) or the other analysed peptides.

### 2.2. Hydrogen bonding and supramolecular aspects

When discussing crystal architecture, it is worthwhile to focus on both intra- and intermolecular H-bonding interactions. The molecular packing of (**1**) in the solid state is mainly influenced by intermolecular (O-H^…^O, N-H^…^O, C-H^…^O) and intramolecular (N-H^…^O, N-H^…^N, C-H^…^O and C-H^…^N) hydrogen bonding (in the range 1.88(3)–2.568(19) Å for the H^…^A distance). Similar H-bonding interactions were observed for the other crystals analysed. The prominent intra- and intermolecular H-bonding interactions associated with (**1**) and other structures are shown in table 1 and the electronic supplementary material, table S2, respectively. In (**1**), molecules are linked to one another via classical hydrogen bonds in a stacking pattern, which is a common phenomenon in peptides with folded conformations [51]. It is also clearly visible in the other analysed structures, that is JOVWEW and BEFVEQ01. These crystals are the most compactly packed crystal structures, with Kitaigorodskii Packing Index (K.P.I.) (values of 71 [52].

**Table 1.** Geometrical parameters of hydrogen bonds in (**1**). *, intramolecular interactions; D, donor; A, acceptor.

D-H^…^A	D-H	H^…^A	D^…^A	D-H^…^A
*N1-H1^…^O2	0.860(3)	2.114(3)	2.566(3)	112.3(2)
*N1-H1^…^N2	0.860(3)	2.286(3)	2.730(3)	112.2(2)
O1-H1A^…^O3^i^	0.82(2)	1.88(3)	2.692(3)	173(2)
N2-H2^…^O5^ii^	0.84(2)	2.10(3)	2.931(3)	171(2)
*C5-H5A^…^O3	0.970(4)	2.515(3)	3.060(3)	115.5(3)
*C6-H6A^…^N1	0.961(11)	2.568(19)	2.943(4)	103.4(16)
*C10-H10B^…^O4	0.961(16)	2.444(12)	3.047(3)	120.6(10)
*C12-H12^…^O5	0.980(3)	2.398(3)	2.803(3)	104.1(2)
C14-H14A^…^O2 ^iii^	0.959(7)	2.473(14)	3.140(4)	126.5(15)
symmetry codes:				
(i) *1 − x, 1/2 + y, 1/2 − z;* (ii) *−1/2 + x, 1/2 − y, −z;* (iii) *1 + x, y, z*				

In (**1**), the molecules pack in a head-to-tail manner that runs antiparallel along the *b-*axis. The molecules are linked together by O1-H1a^…^O3 and N3-H3^…^O3 hydrogen bonds along an *a*-axis, forming helical supramolecular chains at the first level of graph-set theory, which are denoted as *C*(7) and *C*(8), respectively.

**Figure 4.**
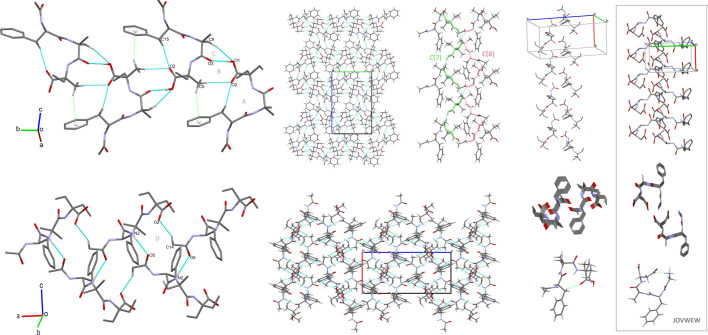
The supramolecular self-assembly of (**1**), showing the following H-bonding motifs: A – *S*(11), B – *R*^2^_2_(10), C – *R*^2^_2_(7), D – *R*^3^_3_(19). The structure is stabilized by weak C-H^…^π interactions.

Consequently, cyclic motifs (i.e. *R*^3^_3_(19)) are formed at the second level of the supramolecular architecture, which indirectly promotes the folded conformation. The crystal packing of (**1**) is presented in figure 4, and the crystal packing of all analysed structures is shown in the electronic supplementary material, figure S3. The oxygen atom O1 is either a donor or acceptor, participating in the O1-H1A^…^O3 (−x, ½+y) and C9-H9C^…^O1 interactions, respectively. Consequently, the *R*^2^_2_(7) supramolecular cyclic motif was formed. The O3 and O4 atoms are bifurcated acceptors, whereas O2 is a trifurcated acceptor. They all participate in forming various supramolecular synthons at the first and second levels of the supramolecular architecture.

For comparison, bi- and trifurcated acceptors were also observed in JOVWEW and BEFVEQ01, whereas only bifurcated acceptors were observed in HOTYAR and VISYII. In addition, bi- or trifurcated donors were also visible in JOVWEW. Notably, bifurcated H-bonding motifs are common in this class of compounds. The new synthons are shown in the electronic supplementary material, figure S4. A noteworthy observation is that the *R*^2^_2_(10) motif in BEFVEQ01 is only a centrosymmetric supramolecular pattern. The library of supramolecular patterns observed in all analysed crystals is shown in the electronic supplementary material, table S3.

**Figure 5.**
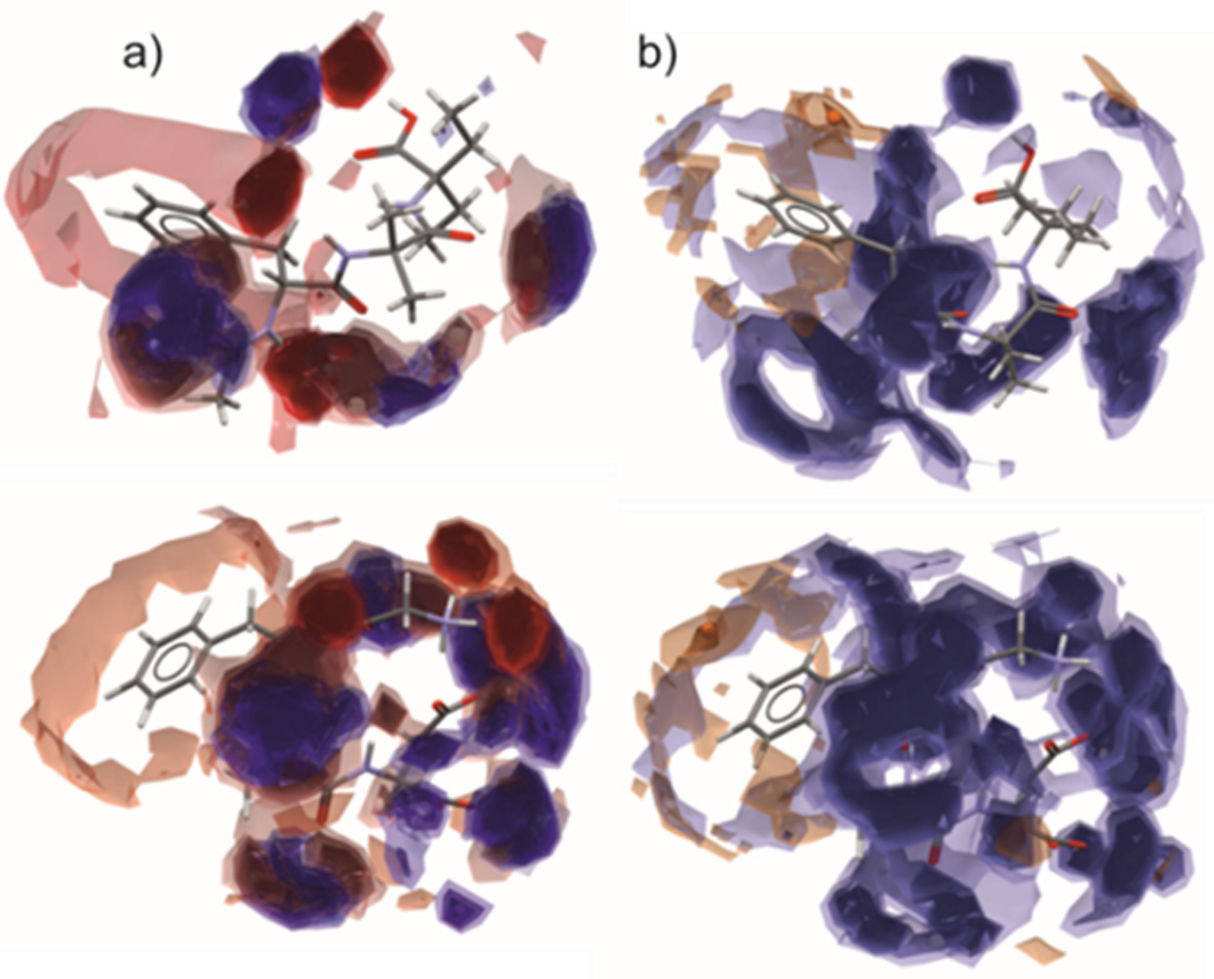
Full interaction maps representing the regions of higher probability of interactions with (a) NH donors (blue) and O acceptors (red). (b) Hydrophobic (orange) and hydrophilic (blue) regions of (**1**) (top) and JOVWEW (bottom) molecules in crystals.

Statistical distributions derived from the extensive collection of structures in the CSD allow representation of the most likely locations of selected functional groups in the form of full interaction maps (FIMs). As expected, the preferential H-bond donors (blue areas in figure 5a) and acceptors (red regions in figure 5a) appeared near the peptide and carboxylic groups for both (**1**) and JOVWEW molecules. In particular, carbonyl groups tend to interact with different NH donors and OH groups. Noticeably, in the conformation of molecule (**1**) in the crystal, the N1H donor is not likely to interact at all. Figure 5b shows only the position of the most favourable area for interactions with the aromatic C-H and water oxygen atom probes. These may be good indicators for depicting the hydrophobic and hydrophilic nature of the analysed molecules. In the case of (**1**) and JOVWEW, a distinct separation of these regions occurs. Moreover, the hot spot (orange ball) above the phenyl ring indicates that the molecule is likely to be involved in aromatic π^…^π interactions. However, the Aromatic Analyzer calculations revealed only weak contacts in the case of (**1**), whereas strong π^…^π and moderate C-H^…^π interactions were detected in JOVWEW, (electronic supplementary material, figure S5).

**Figure 6.**
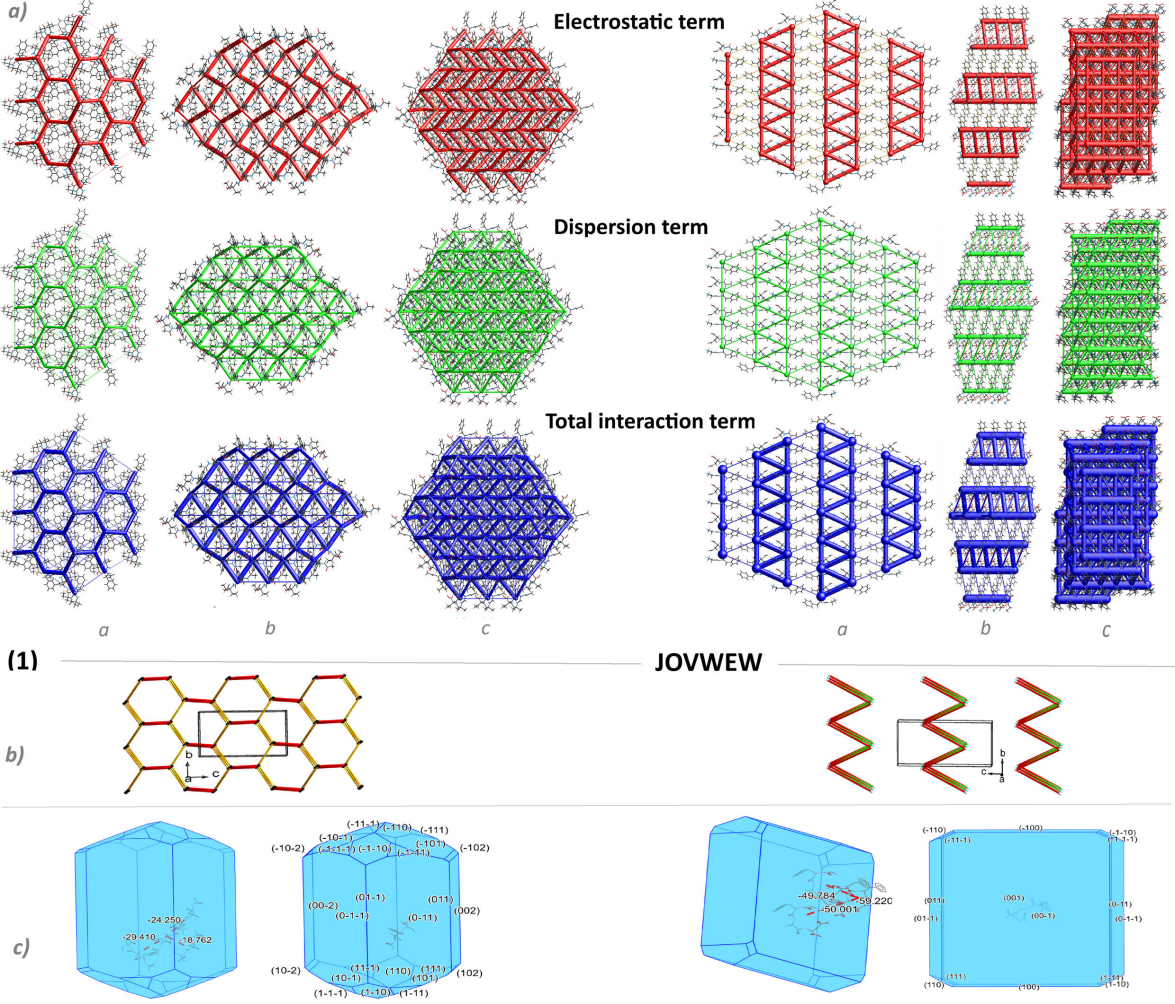
(*a*) Perspective views of the energy frameworks (EF) calculation (electrostatic, dispersion and total interaction energies) for a cluster of nearest-neighbour molecules in (**1**) and JOVWEW (the energy tube size is 100, and the energy threshold is 0 kJ mol^−1^); (*b*) The topology of the most energetically favourable intermolecular interactions related to LSAMs in (**1**) and JOVWEW. The molecules are reduced to a node (black balls) and the lines between them represent the directionality of the intermolecular interactions; (*c*) The calculated (VisualHabit) morphology of the crystals of (**1**) and JOVWEW with the indication of the strongly interacting molecules (the energy of interactions in kJ mol^−1^) and facets (hkl) indices).

The supramolecular assembly of (**1**) and its folded conformation are supported by weak C-H^…^π interactions (figure 4). C-H^…^π contacts also stabilize the supramolecular systems in HOTYAR and VISYII. Relatively significant π^…^π, C-O^…^π and C2-Cl4...π interactions were observed in VISYII (electronic supplementary material, tables S4 and S5). Mercury’s hydrogen bond propensity (HBP) module ranks hydrogen bond donors and acceptors and generates network landscapes. In crystals, (**1**) molecules form three hydrogen bonds, which use all the best donors (rank 1−3) and first- and third-rank acceptors, and the O3 amide acts as a bifurcated acceptor (electronic supplementary material, figure S6). In the network landscape, the best HBP is achieved by employing the three best donors and acceptors, and where the same rank donor and acceptor form the interaction: O1H^…^O5, N3H^…^O4 and N2H^…^O3. The best coordination is achieved when four hydrogen bonds are formed, that is, an additional bond is formed by N1H and O2 (both rank 4). Interactions calculated for the TBXA2R protein also revealed three H-bonds with the molecule of (**1**), and all three best acceptors were engaged in hydrogen bond interactions (see section below). For CCR5 and Lysophosphatidic Acid Receptor 1 (LPA1), four and five H-bonds were found, respectively, which may be related to better H-bond coordination; however, bifurcated donors and acceptors might weaken the interactions with the protein. In the CSD-particle module, the VisualHabit tool [53] was used to calculate the crystal morphology based on the crystal structure and determine intermolecular interactions. For the latter, various force fields can be used. In our case, we chose the Dreiding II force field with Gasteiger charges and a limiting radius of 30 Å. First, the calculated energy of the three most critical intermolecular interactions is significantly smaller than that obtained by the energy frameworks calculations. However, their motives are the same. Remarkably, the plate shape of the JOVWEW crystal is in agreement with the intermolecular interactions topology (figure 6).

Figure 6 illustrates modular large synthons, known as long-range synthon Aufbau module (LSAM) [54], and the intermolecular contact topology, where each molecule is represented as a node. The lines connecting these nodes corresponded to the observed H-bonds and significant aromatic interactions. In the case of (**1**) (left), a three-dimensional network is observed, which can be described as a semi-regular network with vertex 6 (46.423.646) and the Reticular Chemistry Structure Resource (RCSR) reference *acs* (http://www.rcsr.net/nets/acs).

In JOVWEW, the two-dimensional network was observed with vertex 6 (36.66.823) having a distorted *hxl* in the RCSR database (http://www.rcsr.net/layers/hxl).

### 2.3. Hirshfeld surface analysis

**Figure 7.**
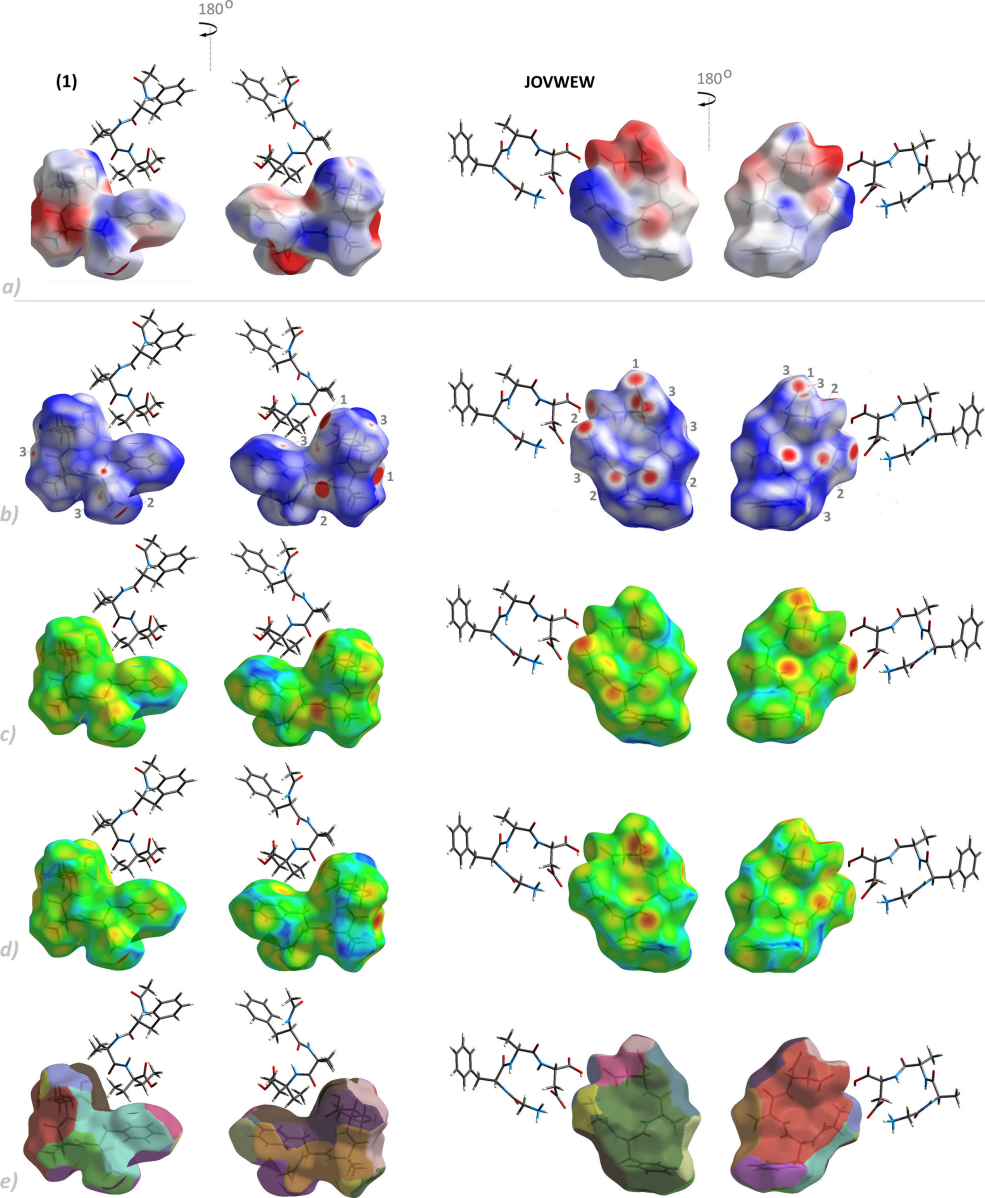
Hirshfeld surfaces (front and back view) of (**1**) and JOVWEW drawn over (*a*) *d_norm_*, (*b*) *d_i_*, (*c*) *d_e_* and (*d*) *fragment patch* (1 - O-H^…^O, 2 N-H^…^O, 3 C-H^…^O). The surfaces are shown transparent to allow visualization of molecules.

HS analysis was performed to obtain a deeper insight into the intermolecular interactions contributing to crystal formation. The HS maps of the single-crystal structures were calculated for *d*_norm_, *d_i_*, *d_e_* and fragment patches (figure 7; electronic supplementary material, figure S7) in two orientations (front and back views). On the *d*_norm_ surfaces, the red and blue areas signify shorter and longer contacts, respectively, compared to the van der Waals radii. At the same time, the white regions indicate contacts equal to the sum of the van der Waals radii of atoms. The red spots indicate the existing primary intermolecular forces. To be more precise, the red areas labelled 1 and 2 are attributed to O-H^…^O and N-H^…^O hydrogen bonds, respectively. The smaller circular red regions labelled 3 are assigned to intermolecular contacts such as C-H^…^O.

Blue areas indicate H–H interactions. The *d_e_* and *d_i_* surfaces describe the distance from the HS to the closest nuclei inside and outside the surface, respectively. The *fragment patch* property helps calculate coordination numbers. Thus, we can determine the number of molecules interacting with a central moiety and find the closest external main molecular fragments.

HS analysis was extended to characterize the potential electrostatic interactions between molecules and their neighbouring moieties. It concerns both the crystal (supramolecule) and the bio-system (bio-supramolecule). The latter is primarily related to active sites.

The molecular electrostatic potentials mapped over the HS identify the reactive sites of the analysed molecules for electrophilic or nucleophilic attack. The red spots indicate the most significant negative charge, making it more susceptible to electrophilic addition. The blue zones denote the most critical positive charge, indicating a favourable site for nucleophilic attack. Hydrophobic interactions in aromatic rings indicate potential interactions with protein targets. Furthermore, intramolecular interactions (C-H^…^O in (**1**) and N-H^…^O in JOVWEW) may reduce the negative charge in the middle area of the moieties, which is more clearly visible in JOVWEW owing to stronger N-H^…^O interactions and consequently better stabilization of the folded conformation.

**Figure 8.**
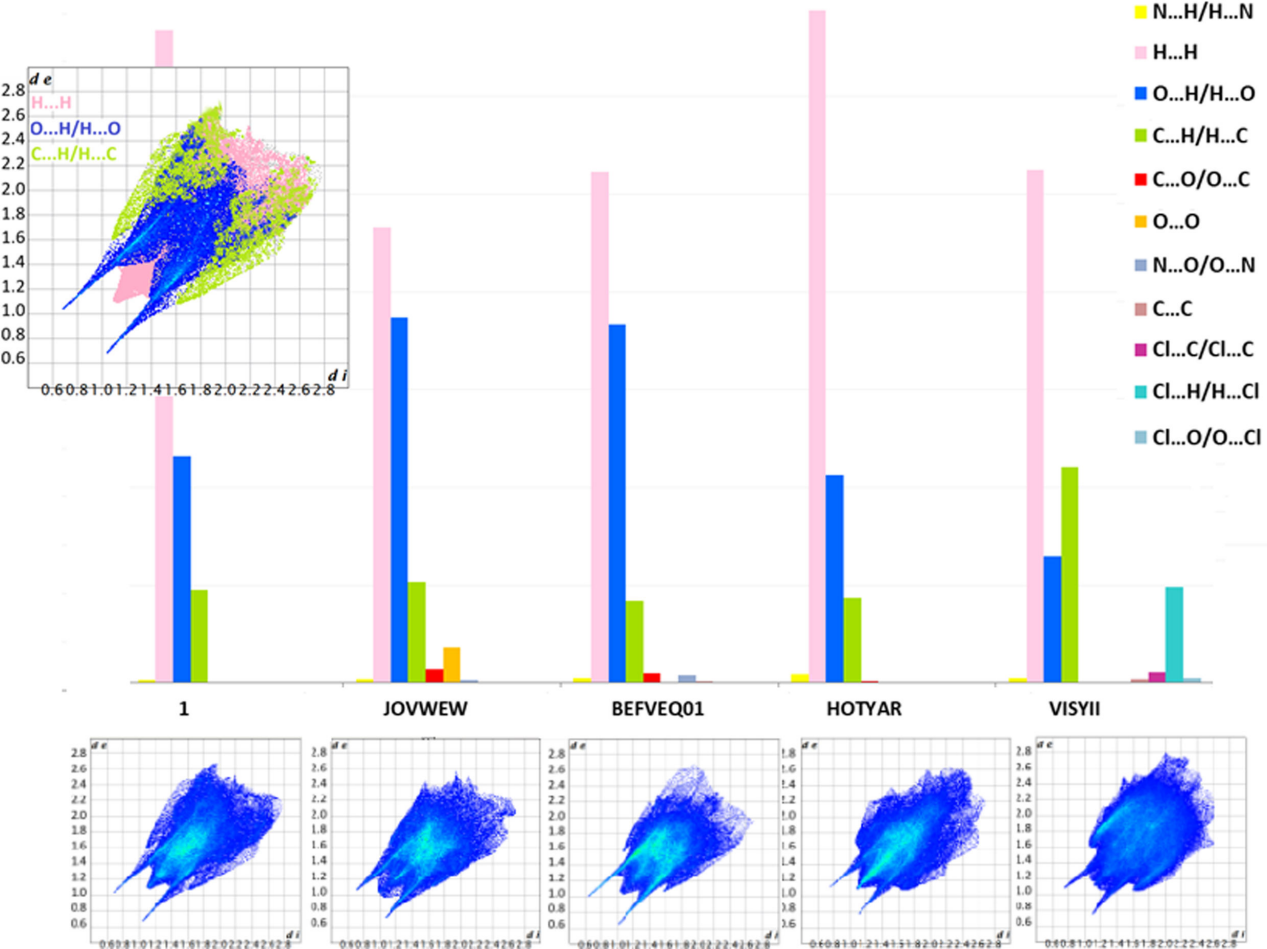
The contribution of close inter-contacts in the HSs of analysed crystals. Fingerprint Plot (FP) diagrams for all compounds and the two-dimensional decomposed FP for (**1**).

Further analysis revealed that the most significant contributors to HS were the H^…^O/O^…^H, H···C/C···H and non-bonding H···H interactions. Nearly all structures, apart from VISYII, prefer O...H/H...O over C...H/H...O interactions. The opposite situation occurs in VISYII. It is noteworthy that the two-dimensional fingerprint plot of (**1**) is dominated by a pair of strong symmetric spikes corresponding to the O^…^H/H^…^O distances (figure 8), highlighting the key role of the carboxylic–carboxylic and amide–amide interactions. Furthermore, Cl^…^H/H^…^Cl in VISYII and O^…^O and C^…^O/O^…^C in JOVWEW are also relevant. Other identified interactions, i.e. Cl^…^C/C^…^Cl in VISYII, N^…^O/O^…^N in BEFVEQ01, N^…^H/H^…^N in HOTYAR, BEFVEQ01 and VISYII, Cl^…^C/C^…^Cl, and π^…^π in VISYII are insignificant contributors to the total HS (below 1 %).

To understand the supramolecular energy networks responsible for the crystal architectures, energy framework calculations were carried out, with energy components described as *E*_ele_ (electrostatic/coulombic energy), *E*_rep_ (repulsion energy), *E*_disp_ (dispersion energy) and *E*_pol_ (polarization energy).

As reported by Mackenzie *et al.* [55], the scaling factors for generating electron densities in figure 6 and the electronic supplementary material, figure S8 present the three-dimensional topology of the interactions between molecular pairs in (**1**) and other analysed crystals, respectively. It illustrates the direction and magnitude of the interactions across their energy components.

The interaction energies are visualized by cylinders connecting the molecules, where the cylinder radius is proportional to the interaction strength. The analysis revealed that in (**1**), HOTYAR and VISYII, the dispersion energy attributed to weak close interactions predominates over the electrostatic energy and significantly contributes to the total interaction energy. Only in (**1**) repulsion energy makes a contribution similar to the dispersion part. In JOVWEW and BEFVEQ01, the electrostatic interactions resulting from hydrogen bonding significantly contributed to the intermolecular framework (electronic supplementary material, table S6).

### 2.4. Density functional theory study

**Figure 9.**
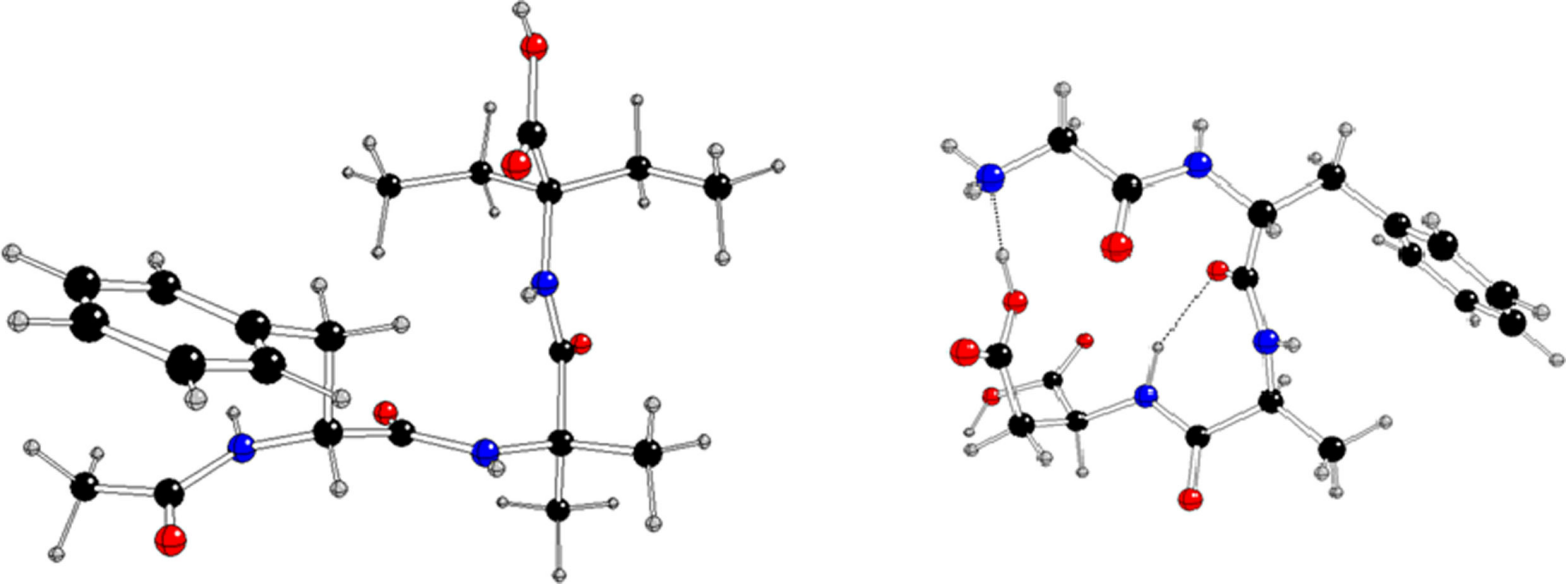
DFT optimized structure of (**1**) (on the left) and JOVWEW (on the right) in aqueous solution (C – black, O – red, N – blue, H – grey).

We focused on the common parts of compounds (**1**), BEFVEQ01 without an aqueous molecule (here denoted BEFVEQ01x), HOTYAR and JOVWEW from the CSD [56]. Geometry optimization was based on X-ray structures. The DFT-optimized structures in water are presented in figure 9 and the electronic supplementary material, figures S9 and S10.

Our comparative study suffered from different atom numbering in the studied compounds. Therefore, we introduced atom notation as shown in the electronic supplementary material, figure S11 and table S7.

As BEFVEQ01 was optimized without a solvent molecule present in the X-ray structure of BEFVEQ01, the hydrogen bond between the side chain R_8_ = -CH_2_-C(O)OH and water oxygen was toggled to the phenoxyl oxygen of the side chain R_4_ = -CH_2_-(p-Ph-OH). Consequently, the distance between both oxygen atoms (in R_8_ and R_5_ chains) was reduced from 3.093(3) Å in the X-ray structure to 2.765 Å and 2.756 Å in the DFT structures optimized in vacuum and aqueous solutions, respectively.

The X-ray JOVWEW structure corresponds to a zwitterion with R_2_ = -NH_3_^+^ and R_8_ = -CH_2_-C(O)O^-^ with N1-H1...O4 hydrogen bonding between them. However, during DFT geometry optimization this hydrogen bond is changed to N1...H1-O4 (figure 9), and the zwitterionic form ceases to exist (R_2_ = NH_2_ and R_8_= -CH_2_-C(O)OH). On the other hand, the X-ray structure contains another N1-H2...O4‘ hydrogen bonding with the O4’ atom of the neighbouring molecule which is missing in the DFT optimized structures of a single JOVWEW molecule. The zwitterionic form seems to be stabilized by the simultaneous existence of hydrogen bonds N1-H1...O4 and N1-H2...O4. The relevant interatomic distances in the JOVWEW subsystems are listed in the electronic supplementary material, table S8.

**Figure 10.**
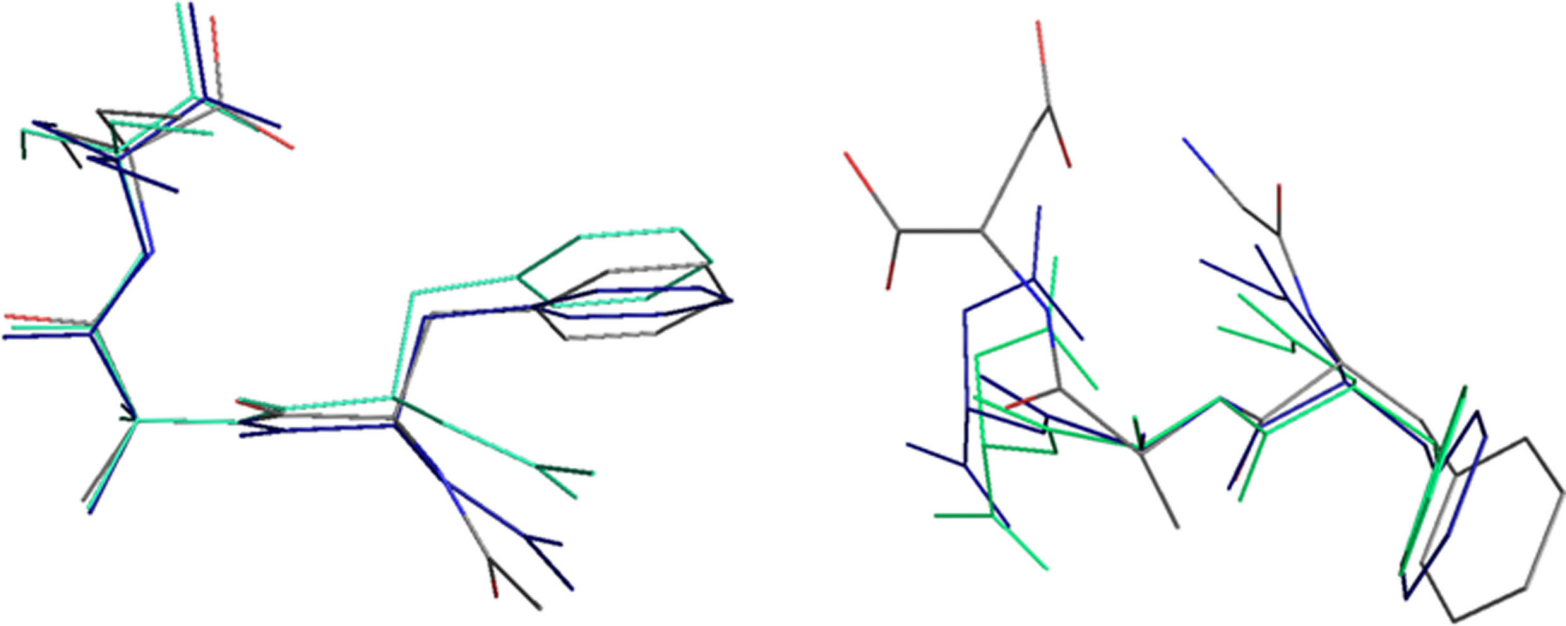
Overlay of the X-ray structure of (**1**) (on the left) and JOVWEW (on the right) (C—grey, N—blue and O—red) and of its DFT optimized structures in vacuum (blue) and in aqueous solution (green) with respect to N_f_, C_g_ and H_f_ atoms. H atoms are omitted for clarity.

The overlay of the X-ray structure of the compounds under study and the DFT-optimized structures in vacuum and aqueous solutions are shown in figure 10 and the electronic supplementary material, figures S12 and S13. The differences between them can be explained by the ordered environment defined by other molecules in the crystal, such as packing forces and intermolecular hydrogen bonds. A quantitative measure of the similarity between two or more structures is the root mean square deviation (RMSD) of the corresponding atomic positions (i.e. the average distance between the corresponding atoms of superimposed molecules). The data in the electronic supplementary material, table S9 confirm that the similarity between the X-ray structures and both DFT optimized structures is significantly lower than that between the DFT-optimized structures in vacuum and aqueous solution.

**Figure 11.**
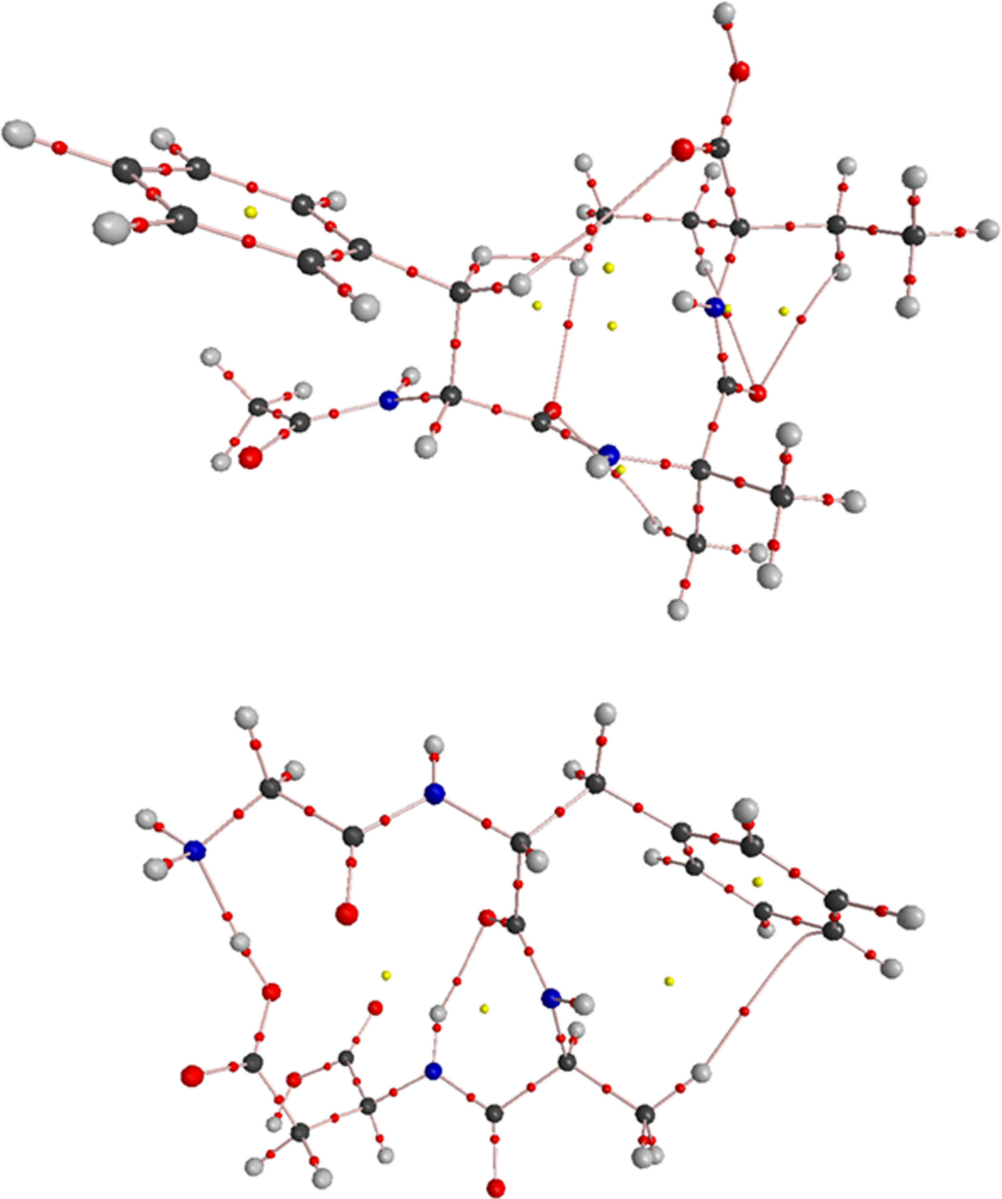
Molecular graph of (**1**) (top) and JOVWEW (bottom) in aqueous solution (C–black, O–red, N–blue, H–grey, bond critical points–small red, ring critical points–yellow).

The largest differences in the DFT-optimized and solid-state geometries were observed mainly for the torsion angles (electronic supplementary material, table S10). Nevertheless, the C_a_-C_b_-N_c_-C_d_, C_e_-N_f_-C_g_-C_h_ and C_g_-C_h_-N_i_-C_j_ torsion angles (of the C-C_O_-N-C type, where C_O_ is the C atom bonded to O) do not differ significantly and correspond to nearly planar conformations (approx. 170^o^) both in the solid state and in aqueous solutions, which is similar that for C_O_ = N double bonds. This is surprising because, except for C = O carbonyl and C-C phenyl bonds, all remaining C-C and C-N bonds are formally single in our structures. This tendency towards planarity cannot be explained by weak interactions such as hydrogen bonds, as indicated by their molecular graphs (figure 11; electronic supplementary material, figures S14, S15 and by O and N atomic charges (table S11). Thus, we will study this problem in terms of Quantum Theory of Atoms-in-Molecule (QTAIM) topological analysis.

The non-bonding intramolecular bond paths in the compounds under study differed significantly. Their number, related to the common part of our compounds varies between 2 and 8 (compare the electronic supplementary material, table S11); therefore, these interactions cannot explain the almost planar C-C_O_-N-C structures mentioned above. As expected, the O atoms have more negative charges than the N atoms, whereas the C and H atoms have positive charges. The atomic charges are only slightly affected by non-bonding intramolecular interactions.

Single and double bonds can be distinguished according to their lengths; however, this problem is usually more complicated (electronic supplementary material, table S12). Very good agreement between the X-ray and DFT-optimized structures in aqueous solutions can be concluded for the bond length of non-H atoms (despite different environments) in our systems. Although all C=O bond lengths are equal to *ca* 1.2 Å, the N-C bonds can be divided into two groups. The lengths of the C_b_-N_c_, C_e_-N_f_ and C_h_-N_i_ bonds are approximately 1.3 Å, whereas those of N_c_-C_d_, N_f_-C_g_ and N_i_-C_j_ were approximately 1.5 Å. Single C-C bond lengths are *ca* 1.5 Å long as well. The single C_k_-O_l_ bond lengths (*ca* 1.3 Å) are longer than the double C=O bonds.

The character of the individual bonds in our DFT-optimized structures in aqueous solutions was studied in terms of the QTAIM topological analysis of the electron density (electronic supplementary material, tables S13–S17). All formally double C=O bonds have delocalization indices (DI) of approximately 1.2 (typical for double bonds), bond critical point (BCP) electron density *ρ*_BCP_ approximately 0.40 e/Bohr^3^, BCP Laplacian ∇^2^*ρ*_BCP_ approximately −0.4 e/Bohr^5^ and BCP ellipticity *ε*_BCP_ approximately 0.04 (too small for double bond). Formally single N-H bonds have a DI of approximately 0.7 (typical for single bonds), *ρ*_BCP_ approximately 0.34–0.35 e/Bohr^3^, ∇^2^*ρ*_BCP_ approximately −1.9 e/Bohr^5^ and *ε*_BCP_ approximately 0.07. The C_b_-N_c_, C_e_-N_f_ and C_h_-N_i_ bonds have DI approximately 1.0 (except for C_b_-N_c_ in BEFVEQ01x), *ρ*_BCP_ approximately 0.33–0.34 e/Bohr^3^, ∇^2^*ρ*_BCP_ approximately −1.0 e/Bohr^5^ and *ε*_BCP_ approximately 0.2 (typical for double bonds). The N_c-_C_d_, N_f_-C_g_ and N_i_-C_j_ bonds have DI approximately 0.9, *ρ*_BCP_ approximately 0.26–0.27 e/Bohr^3^, ∇^2^*ρ*_BCP_ approximately −0.7 e/Bohr^5^ and *ε*_BCP_ approximately 0.03–0.05, respectively, and these characteristics are more similar to single C-C bonds. All the bonds in our molecules have negative ∇^2^*ρ*_BCP_ values that are typical for covalent bonds.

**Figure 12.**
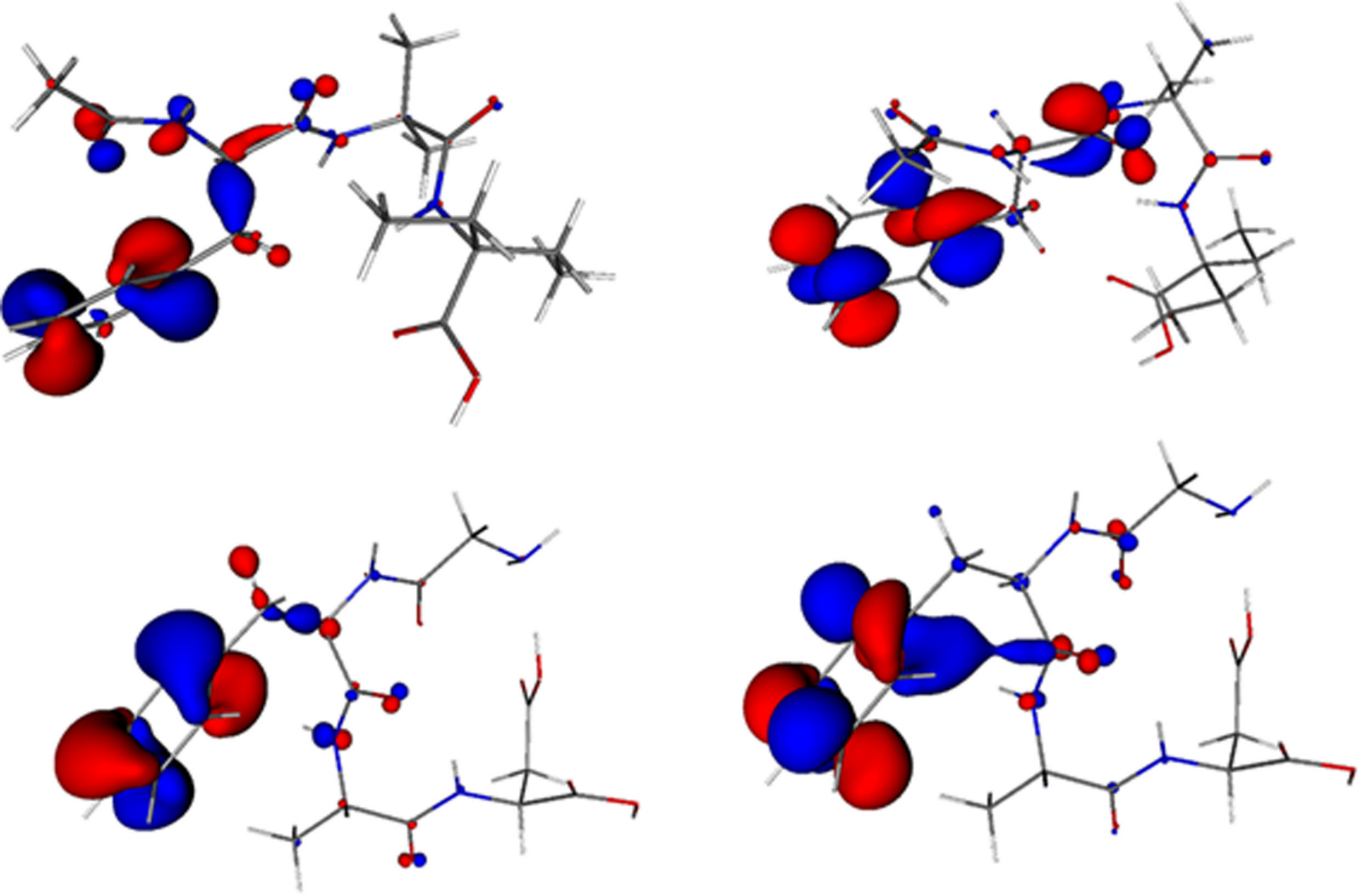
HOMO (left) and LUMO (right) of (**1**) (top) and JOVWEW (bottom) in aqueous solutions (0.05 a.u. isosurface).

To gain deeper insight into the electronic characteristics of the analysed molecules, frontier molecular orbitals, such as the highest occupied molecular orbital (HOMO) and the lowest unoccupied molecular orbital (LUMO), were analysed. According to Fukui rules [57,58], the HOMO is viewed as nucleophilic or electron donating, whereas the LUMO is electrophilic and electron accepting. The energy gap between the HOMO and LUMO in the studied compounds was greater than 6 eV, indicating their stability (electronic supplementary material, table S18). The negative LUMO energies indicated a higher affinity for reduction. Both HOMO and LUMO (figure 12; electronic supplementary material, figures S16, S17) are located mainly at the phenyl rings and to a significantly lower extent at some heteroatoms (such as O_b_,N_c_, O_e_ and N_f_). Electrophilic and nucleophilic reagents can attack C_d_ and C_e_ sites, respectively.

### 2.5. Absorption, distribution, metabolism and excretion-toxicity and beyond

Apart from BEFVEQ01, nearly all peptides were potentially bioactive, with a peptide ranker score above 0.65.

Physicochemical and bio-pharmacokinetic properties, including absorption, distribution, metabolism and excretion (ADME) profiles, were predicted using the web-based SwissADME platform, and the results are presented in the electronic supplementary material, table S19 and figures S18, S19, respectively.

The molar refractivity falls within the acceptable range (30–140) for (**1**), JOVWEW and BEFVEQ01.

The topological polar surface area (TPSA) is crucial for drug candidates, as it strikes a balance between lipophilicity and hydrophilicity, ensuring optimal efficacy and safety.

The TPSA value of 124.60 Å^2^ for (**1**) suggests that this molecule may have better oral bioavailability, as it may cross cell membrane through passive diffusion, than other analysed compounds with higher TPSA values.

The lipophilicity values (especially iLogP and SILICOS-IT) were favourable for nearly all molecules, apart from VISYII (the acceptable range from −0.4 to +5.6). The calculated values of the LogS parameter, characterizing drug absorption and distribution, indicate satisfactory water solubility of the analysed molecules. The exception was VISYII. The predicted gastrointestinal (GI) absorption was high only for (**1**). Drug-likeness, a factor of oral drug potential, is satisfied only for (**1**) with one violation (Veber). Moving forward, a bioavailability score above 50% has only (**1**).

To conclude, (**1**) exhibited the best pharmacokinetic characteristics among all the analysed compounds regarding drug-likeness, including bioavailability. Therefore, we supplemented the information for (**1**) using Deep-PK, the newest deep-learning-based pharmacokinetics (PK) and toxicity prediction platform [59]. AMES tests related to mutagenic potential and human Ether-à-go-go-Related Gene (hERG) I and II, which assess the risk of ventricular arrhythmia yielded satisfactory results. Moreover, no skin sensitization was observed. However, it is also predicted to be hepatotoxic. Detailed results are provided in the electronic supplementary material, table S20. According to the Pro-tox II platform, (**1**) belongs to the toxicity class 5.

The probable activity of the analysed molecules was calculated *via* the Prediction of Activity Spectra (PASS) for Substances web server. One of the potential activities (with *Pa* values greater than approx. 0.8) is the saccharopepsin inhibitor activity for all analysed molecules, apart from VISYII. Saccharopepsin (proteinase A) is associated with, among other things, neoplastic diseases [60].

All molecules exhibited potential cytotoxicity towards cancer cell lines, as determined by the CLC-Pred web platform (electronic supplementary material, table S21).

Computational assessments via CLC-Pred [61] indicated that (**1**) exhibits promising anticancer potential with predicted activity against several cancer-related pathways and cell lines, including cisplatin-resistant ovarian carcinoma (A2780cisR), breast adenocarcinoma (MDA-MB−468), non-small cell lung carcinoma (NCI-H460) and breast carcinoma (T47D).

**Figure 13.**
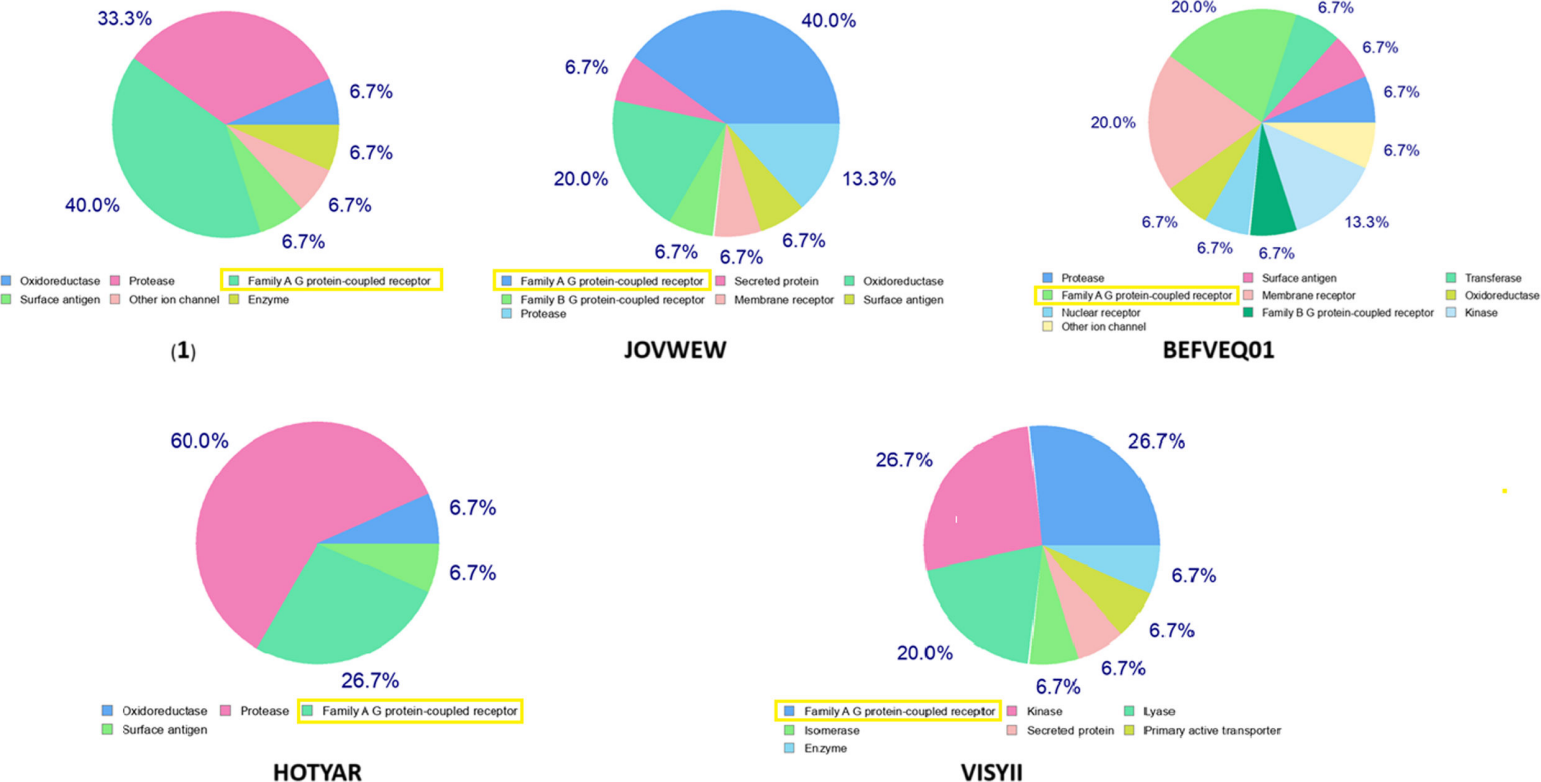
Predicted biological targets using SwissTarget platform for analysed compounds.

According to the SwissTargetPrediction service, all molecules are potential ligands for the A family of GPCR proteins (figure 13), with (**1**) exhibiting the best pharmacokinetics.

Our future studies will focus on applying molecular optimization and docking studies to improve the bioactivity of similar compounds towards A family GPCRs.

### 2.6. Docking study

To validate the potential anticancer activity of the analysed molecules in terms of GPCRs, we performed a molecular docking analysis against the corresponding targets associated with various types of cancer. Docking is an advanced computational technique used to predict the structure of ligand-receptor complexes by estimating the strength of non-covalent interactions within the active site.

*In silico* experiments were performed to assess the binding affinities between four small molecules ((**1**), BEFVEQ01, HOTYAR and JOVWEW) and a group of GPCRs involved in cancer pathways. We can note that VISYII was excluded from the analysis because the results from the two software were inconsistent and unacceptable.

The chosen GPCRs, from the Protein Data Bank (PDB) [62], comprised ADRB2, CCR5, CXCR4, FPR1, LPR1, OPRD1, OPRM1, PAR1, PTGER4, S1PR1 and TBXA2R, as determined through molecular docking via Autodock 4.2.6 (electronic supplementary material, table S22).

**Figure 14.**
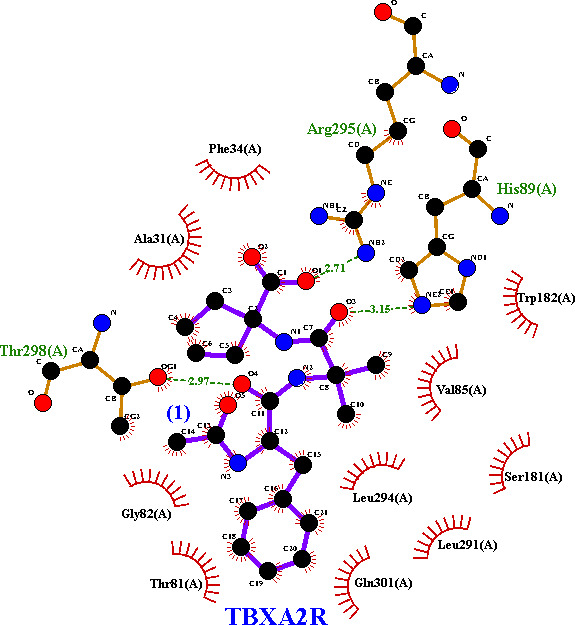
LigPlot representation of the interaction between (**1**) and TBXA2R. In this interaction, His89, Arg295 and Thr298 play active roles, forming three hydrogen bonds with the ligand. The violet solid lines represent ligand bonds, while black solid lines depict non-ligand bonds. Green dashed lines with numerical values indicate hydrogen bonds and their respective lengths. Red arcs labelled with specific residues highlight non-ligand residues involved in hydrophobic interactions, while black circles mark the atoms participating in these interactions.

Among the four tested small molecules, TBXA2R exhibited the strongest binding affinity, particularly with (**1**) (−7.59 kcal mol^−1^) and BEFVEQ01 (−5.85 kcal mol^−1^). (**1**) forms three hydrogen bonds with His89, Arg295 and Thr298, highlighting a strong interaction profile (figure 14). Similarly, BEFVEQ demonstrated stable interaction with TBXA2R, forming four hydrogen bonds involving His98, Thr29 and Arg295 (electronic supplementary material, figure S20). TBXA2R could be a key target for these ligands given their favourable binding scores and hydrogen-bonding networks.

**Figure 15.**
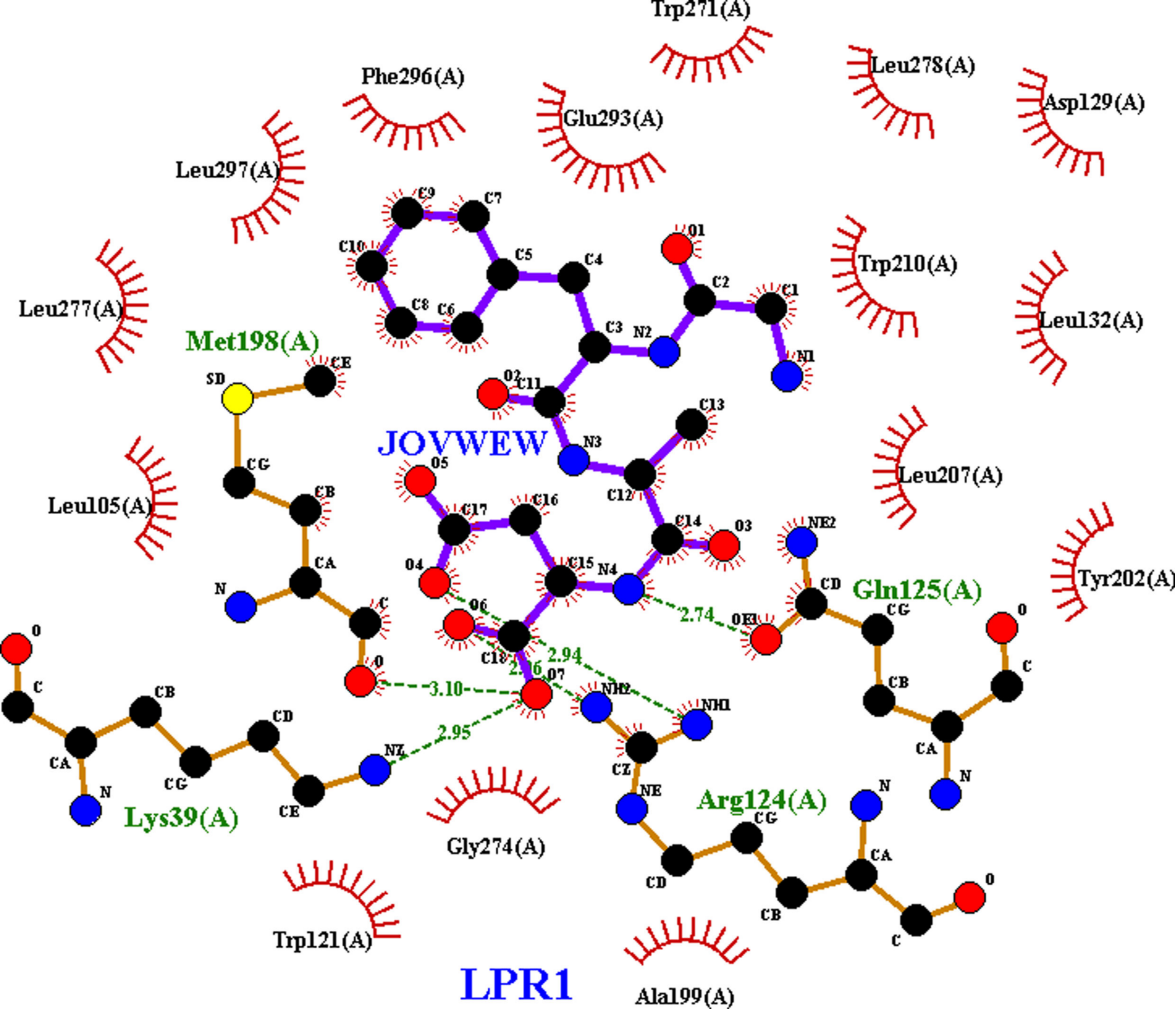
LigPlot representation of the interaction between JOVWEW and LPR1. The violet solid lines represent ligand bonds, while black solid lines depict non-ligand bonds. Green dashed lines, accompanied by numerical values, indicate hydrogen bonds and their respective lengths. Red arcs, labelled with specific residues, highlight non-ligand residues involved in hydrophobic interactions, while black circles mark the atoms participating in these interactions.

CCR5 displayed significant ligand binding, with docking scores of −5.59 kcal mol^−1^ to HOTYAR. In addition, JOVWEW had a noticeable binding score for LPR1, −5.65 kcal mol^−1^ and four hydrogen bonds, which made it stand out among other proteins (figure 15). The interaction of CCR5 with HOTYAR is illustrated in the electronic supplementary material, figure S21, where the key residues contribute to ligand stabilization within the binding site.

CXCR4, a key chemokine receptor in tumour metastasis, showed relatively weaker ligand binding, with docking scores ranging from −2.65 kcal mol^−1^ (**1**) to −1.56 kcal mol^−1^ (JOVWEW). Similarly, PAR1 and PTGER4 showed lower binding affinities, particularly with HOTYAR (−2.8 kcal mol^−1^ for PAR1 and −0.48 kcal mol^−1^ for PTGER4), suggesting minimal receptor engagement.

FPR1 and LPR1 showed moderate binding affinities, with (**1**) scoring −4.46 kcal mol^−1^ for FPR1 and −5.01 kcal mol^−1^ for LPR1, forming one and two hydrogen bonds, respectively. By contrast, the opioid receptors OPRD1 and OPRM1 exhibited moderate to weak interactions with all the tested compounds, with docking scores not exceeding −4.86 kcal mol^−1^.

Overall, these findings identified TBXA2R and LPR1 as the most promising GPCR targets, as both receptors demonstrated the highest binding affinities with three of the tested small molecules. In particular, (**1**) and BEFVEQ have emerged as potential lead compounds, exhibiting strong interactions across multiple targets. These results provide a solid foundation for further experimental validation and optimization of drug development.

The type of binding and residues involved in the interaction are provided in the electronic supplementary material, tables S23–S26 and figures S22–S26.

As shown in the electronic supplementary material, table S27, the conformation varied significantly across different GPCRs, ranging from fully extended to locally folded geometries. It suggests structural flexibility, allowing the peptides to adapt to the unique shape and depth of the binding pocket of each receptor. For example, (**1**) adopted an extended pose when docked with ADRB2; however, it displayed a folded conformation inside the narrower pocket of CXCR4 and FPR1, indicating a potential chameleon-like binding behaviour.

The observed folded or extended conformations reflect not only the geometry of the ligand-binding site of the receptor, but also the physicochemical complementarity, including electrostatic, hydrogen bonding and hydrophobic interactions. Such conformational adaptability is a favourable property in peptide therapeutics, as it helps to improve selective and efficient binding to different structures of GPCR targets [63].

## 3. Conclusions

In this study, we successfully synthesized a novel modified ultrashort peptide, Ac-Phe-Aib-Deg-OH (**1**), using a facile multistep approach. Crystallographic analysis revealed its pseudo-macrocycle nature, ensured by intramolecular interactions, similar to those found in achatin and other derivative ultra-short peptide structures derived from the CSD. A thorough comparative study based on diverse modern computational methods helped explain the pseudo-macrocyclic nature of these peptides, providing valuable insight into their hierarchical supramolecular assembly driven by the interplay of strong and weak interactions. The folded conformation of (**1**) was stabilized by C-H^…^π interactions. It is also indirectly promoted by the formation of larger supramolecular H-bonding motifs, in which multi-furcated donors/acceptors are engaged. A library of supramolecular H-bonding motifs, including new synthons, is provided for this class of compounds. Robust synthons can be used to predict similar self-associating pseudo-macrocyclic ultrashort peptides.

HS analysis revealed that supramolecular assembly is primarily driven by hydrogen bonding, specifically O^…^H/H^…^O and C^…^H/H^…^C. H^…^H contacts are the most significant contributor, and that van der Waals forces are relevant in the stabilization of supramolecular assemblies.

Topological analysis and energy framework calculations showed that dispersion interactions play a central role in the stabilization of the self-assembly of (**1**).

Molecular electrostatic potential analysis revealed strong nucleophilic and electrophilic areas, which are essential for interactions.

DFT results revealed differences between the structures in solution and the solid-state structures, which are influenced by intermolecular interactions, particularly hydrogen bonds.

Different hydrogen bonding patterns in both environments affect the equilibrium between the zwitterionic and neutral structures of JOVWEW.

Nevertheless, these interactions cannot explain the almost planar C-C_O_-N-C structures, which remain independent of the environment. QTAIM analysis shows that their formal single C_O_-N bond is stronger and has a higher π character (i.e. BCP ellipticity) than other N-C and C-C single bonds owing to the lower π character (i.e. ellipticity) of the C=O carbonyl double bonds.

The frontier molecular orbitals (HOMO and LUMO) indicate that phenyl rings, two carbonyl O atoms, two amine N atoms and some neighbouring C atoms are the most probable reaction sites for nucleophilic and electrophilic reactions.

*In silico* bio-pharmacokinetic and pharmacodynamic analyses confirmed that (**1**) exhibits satisfactory drug-like and Absorption, Distribution, Metabolism, Excretion, and Toxicity (ADME-T) properties. The docking results reveal that among the four ligands ((**1**), BEFVEQ, HOTYAR and JOVWEW) tested against the cancer-related GPCRs, (**1**) demonstrated the strongest binding affinities, particularly with TBXA2R (−7.59 kcal mol^−1^) in first rank, then, BEFVEQ also showed notable binding to TBXA2R (−5.85 kcal mol^−1^).

In the third position, JOVWEW showed a noticeable interaction with LPR1 (−5.65 kcal mol^−1^), and HOTYAR exhibited significant binding to CCR5 (−5.59 kcal mol^−1^) in the fourth rank. These findings showed that TBXA2R may be the most relevant target ((**1**), BEFVEQ, as the first rank binding score and second rank with JOVWEW), highlighting its potential as an effective modulator in cancer treatment.

Nevertheless, limitations, such as lack of experimental validation for docking predictions, cannot be neglected. The results underscore the importance of further investigation into ligand–receptor interactions to validate their therapeutic potential in oncological applications.

In summary, (**1**) is an appealing candidate for further optimization and biological testing in the design and development of pseudo-macrocyclic short peptides containing unnatural amino acids as innovative anticancer GPCR ligands with improved pharmacokinetic profiles.

## 4. Material and methods

### 4.1. General chemical synthesis and method for the growth of single crystals

All chemicals used for the synthesis of (**1**) were purchased from commercial sources (Sigma-Aldrich, FLUKA, Merck, Poland). The solvents and substances required for the synthesis and crystallization were used without further purification.

The *N*-protected tripeptide (**1**) Ac-L-Phe-Aib-Deg-OH (Ac: acetyl, Aib: 2-amino−2-methyl propanoic acid, 2-amino-isobutyric acid, alpha-methylalanine, Deg: 2-amino−2,2-diethylethanoic acid, C^α,α^-diethylglycine) was synthesized by stepwise elongation of the C-terminus. The free amino acid H-Deg-OH was obtained using hydantoin from Pentan-d ellipticity ε3-on/potassium cyanide (KCN) [64]. A three-step synthesis of H-Deg-OH t*ert*-butyl esters was developed by K.K. [65,66]. The synthesis of H-L-Phe-Aib-Deg-O*t*Bu was performed following a previously described strategy elaborated and used for C-protected tripeptide H-L-Abu-Deg-Deg-O*^t^*Bu [67]. After acetylation with acetic anhydride/acetic acid solution and splitting of the tripeptide tert-butyl ester with the aid of trifluoroacetic acid, the required final product Ac-L-Phe-Aib-Deg-OH was obtained.

Suitable high-quality single crystals of (**1**) for X-ray data collection were obtained using the vapour diffusion method in a closed system. An optimized mixture of ethanol and water was then prepared.

### 4.2. Single-crystal X-ray data collection and structure refinement

An X-ray diffraction study was carried out on a single crystal of (**1**) using an XtaLAB Synergy Dualflex Pilatus 300 K diffractometer (Rigaku Corporation, Tokyo, Japan) equipped with a charge-coupled device-type area detector, with graphite-monochromated Mo Kα (*λ* = 0.71073 Å) radiation.

The crystal structure was solved using the Olex2 software [68] employing the SHELXT programme for the structure solution and the SHELXL package for refinement [69], which uses least-squares minimization. The non-hydrogen atoms were refined anisotropically. The hydrogen atoms are located in the electron diffraction maps. The structure was refined to an *R* factor of 0.049. The detailed statistics of the crystallization and refinement parameters are listed in the electronic supplementary material, table S1.

Full crystallographic structural data have been deposited at the Cambridge Crystallographic Data Centre (CCDC) with the reference/deposition number **2453414**.

The Mercury 2024.3.1 software and the PLATON programme [70] were employed to perform geometric calculations and generate molecular diagrams, respectively [71].

### 4.3. Computational methodology

#### 4.3.1. Quantum-chemical calculations

Based on DFT, the geometry of the neutral compounds in the singlet ground spin state was optimized using the hybrid functional M06 [72] with GD3 dispersion correction [73] and the cc-pVTZ basis set for all atoms from the Gaussian library [74] in vacuum and in an aqueous solution. Solvent effects were considered by the solvation model based on the solute electron density (SMD) method [75]. The optimized geometries were tested in the absence of imaginary vibrations by vibrational analysis. The Gaussian16 software [74] was used for all quantum-chemical calculations.

The electronic structure of the compounds under study was evaluated using the Quantum Theory of Atoms-in-Molecule (QTAIM) topological analysis of electron density [76]. Atomic charges were obtained by integrating the electron density over the atomic basins. BCPs are saddle points on the bond paths between pairs of atoms, along which the electron density is maximally concentrated. The molecular graph represents a set of bond paths. The BCP electron density *ρ*_BCP_ reflects the bond strength. Its Laplacian, ∇^2^*ρ*_BCP_:


(4.1) $$\nabla^{2}\varrho_{BCP}=\lambda_{1}+\lambda_{2}+\lambda_{3},$$

where *λ*_1_ < *λ*_2_ < 0 < *λ*_3_ are the eigenvalues of the Hessian of the BCP electron density and depicts the relative electron density contribution of the bonded atoms (negative values correspond to covalent bonds). BCP bond ellipticity *ε*_BCP_:


(4.2) $$\varepsilon_{BCP}=\lambda_{1}/\lambda_{2}–1,$$

describes the bond deviation from cylindrical SymmetryMol (such as in ideal single or triple bonds) owing to the double-bond character, mechanical strain or other perturbations.

The electron DI is the average number of electrons delocalized (shared) between pairs of atoms. This corresponds to a bond index if both atoms are connected by a bond path.

QTAIM analysis was performed using AIMAll software [77] .wfn files produced by the Gaussian 16 software. Molecular graphs were constructed using AIM2000 software [78].

The superposition of the optimized structures was optimized using PyMol software, v. 1.8 [79,80]. The RMSD of the corresponding atomic positions of individual pairs of structures were evaluated using the script (https://github.com/charnley/rmsd) [56].

The reactivity of individual molecular active sites against nucleophilic and electrophilic attacks can be predicted based on the HOMO and the LUMO, respectively. Molecular orbitals were drawn using the Molekel software [81].

#### 4.3.2. Docking protocol

##### 4.3.2.1. Protein structure selection and preparation

The three-dimensional structures of selected GPCRs were downloaded from the Uniprot and RCSB PDB [82], with highly resolved structures selected based on experimental validation. Receptors studied include ADRB2(PDB IDs: 2RH1), S1PR1(3V2Y), TBXA2R(8XJN), OPRM1(8EFO), OPRD1(4RWD), PAR1(8XOR), CXCR4(3OE0), FPR1(7VFX), CCR5(7F1S), LPAR1(7TD0) and PTGER4(8GCP) [26,83–93]. Water molecules and ligands bound to them were removed using PyMol or Chimera, and missing loops were modelled using Swiss-Model and AlphaFold as templates [79,94,95].

The structures of the receptors were prepared for docking through the addition of polar hydrogens and assignment of Gasteiger and Kollman charges using MglTools 1.5.7. The receptor and peptide ligand were both converted to the PDBQT format, as required for AutoDock calculation [96].

##### 4.3.2.2. Designing grid box

Grid boxes were defined based on known ligand-binding sites in class A GPCR, including extracellular loops, N-terminal segments and transmembrane domains. For instance, the CCR5 binding site was mapped together with ECL2, ECL3 and TM1, whereas the FPR1 binding site included extracellular loops and an N-terminal domain. The grid box sizes were designed using MglTools to achieve appropriate coverage of the binding pocket while allowing for ligand mobility.

##### 4.3.2.3. Docking simulations and binding energy calculation

Docking simulations were performed using AutoDock v. 4.2.6, where the receptor and ligands in PDBQT format were provided as inputs. Docking was run multiple times to reach consistent position, binding energies (kcal mol^−1^) and the number of hydrogen bonds. These values were useed to identify the most favourable binding position.

##### 4.3.2.4. Analysis and visualization

PyMol or Chimera were then used to analyse the docking experiment results, to visualize the docked peptides at the receptor binding site. LigPlot and Protein–Ligand Interaction Profiler have been used to identify key interactions including hydrogen bonds, and hydrophobic interactions to determine the preferred binding conformation, concentrating on the lowest binding energy conformations [97,98].

### 4.3.3. Mercury 4.0 software use

The CCDC Mercury 4.0 (v. 2024.3.1) programme [71] and its functionalities were used in this study. FIMs, aromatic analysers and HBP modules from CSD materials were used. By contrast, the synthon interaction energies and crystal morphologies were elucidated using CSD-particle functionality [99]. Before any calculation, the molecules were standardized to the CSD conventions concerning the bond types; only nitrogen and oxygen organic donors and acceptors were included for hydrogen bond statistics. For FIMs, standard probes, such as uncharged and charged NH nitrogen groups and RNH_3_, were used to elucidate the spatial preferences for H-bond acceptors. By contrast, carbonyl and alcohol oxygen atoms were used to determine donor preferences. Aromatic CH carbon atoms and water oxygen probes highlight the hydrophobic and hydrophilic sites. All other settings were left to default. The default mode was also sustained for the calculations with the Aromatic Analyzer programme, a neural network-based component that quantitatively assesses aromatic ring interactions within crystal structures.

HBP calculations [100] and hydrogen-bond coordination analysis [101] were performed using the default probes for aliphatic amides and carboxylic groups. The H-bond distance was restricted to not be longer than the sum of the vdW radii plus 0.05 Å. The logistic regression model was trained on 3994 structures with a satisfactory number of valid/false data for all donors and acceptors in structure (**1**). The final area under the receiver operating characteristic curve of 0.83 verifies that the model can be reliably used [102].

### 4.3.4. Hirshfeld surface analysis

HS analysis was performed using CrystalExplorer 21.5 software [103]. The unique HS maps for all analysed specific crystal structures at the molecular level were built around a spherical atom using electron density distribution [104–106]. This approach allows for deeper insight into the intermolecular interactions within the crystals [104]. HS maps for *d_norm_*, *d_i_*, *d_e_* and *fragment patch* properties were generated.

The distances *d_e_* and *d_i_* characterize the distances from the surface to the external nucleus and from the surface to the internal nucleus, respectively. The two-dimensional fingerprint plots combine *d_e_* and *d_i_*, summarizing intermolecular interactions [107].

#### 4.3.4.1. Energy framework analysis

Energy frameworks, including lattice energy and interaction energy analysis, were performed to graphically depict the three-dimensional interaction topology and the nature of the energy related to the interaction present within either the crystal structure of (**1**) or other analysed structures using the CrystalExplorer 21.5 programme, the B3LYP method with the 6−31G(d,p) basis set [103]. The total interaction energy was partitioned into coulombic, polarization, dispersion and repulsion energy contributions. The energy threshold was maintained at a tube size of 150. Only interactions with neighbouring molecules within the coordination sphere at distances of less than 3.8 Å were analysed.

### 4.3.5. Absorption, distribution, metabolism, excretion and toxicity and beyond*—in silico* assay

The PeptideRankerScore software predicted the potential bioactivity of the peptides based on their amino acid composition and sequence (provider: University College Dublin, Ireland [108]). It assigns scores between 0 and 1, with a higher value indicating greater potential activity.

In addition, the PASS web server was used. *Pa* indicates the probability that the compound interacts with the corresponding target. *Pi* reflects the likelihood of adverse outcome [109].

The ADME-T parameters for the analysed compounds were predicted using SwissADME (Molecular Modelling Group of the Swiss Institute of Bioinformatics) [110,111] and the Deep-PK interface [59], a deep learning-based pharmacokinetic and toxicity prediction platform. In addition, the toxicity of the title compounds was evaluated using ProTox-II [112].

Molecular target probabilities, essential to predict phenotypical side effects or possible cross-reactivity, were calculated using the free online tool SwissTarget.

The prediction of tumour (and non-tumour) cell line cytotoxicity was obtained using the CLC-Pred tool based on structure–cell line cytotoxicity relationships using the PASS procedure (prediction of activity spectra for substances) [113].

All ‘in silico’ simulations were performed in May 2025. For calculations, the structures of the analysed compounds were converted into canonical simplified molecular input line entry specifications.

### 4.4. Cambridge structural database survey

A search of CSD, v. 5.46, update of November 2024 [56,114] confirmed the novelty of (**1**). A survey of the most similar compounds yielded four relevant hits, with the following specifications: good quality, no disorder, no polymeric, no powder structures, only organics, no errors, no duplicates and complete three-dimensional coordinates. The CSD reference codes were: JOVWEW [16], BEFVEQ01 [39], HOTYAR [40] and VISYII [41]. A list of the retrieved crystal structures containing the basic crystallographic data is provided in the electronic supplementary material, table S1. Molecular views of the structures are presented in the electronic supplementary material, figure S1.

## Data Availability

Full crystallographic structural data were deposited at the Cambridge Crystallographic Data Centre (CCDC) with reference/deposition number 2453414. These data can be obtained free of charge via https://www.ccdc.cam.ac.uk/structures/ (or from the CCDC, 12 Union Road, Cambridge CB2 1EZ, UK; Fax: +44 1223 336033; E-mail: deposit@ccdc.cam.ac.uk).
